# Supplementary figures and images for: NAA and 6-BA promote accumulation of oleanolic acid by JA regulation in Achyranthes bidentata Bl
Source: PLoS One. 2020 Feb 27;15(2):e0229490. doi: 10.1371/journal.pone.0229490 (PMC7046271; doi:10.1371/journal.pone.0229490)

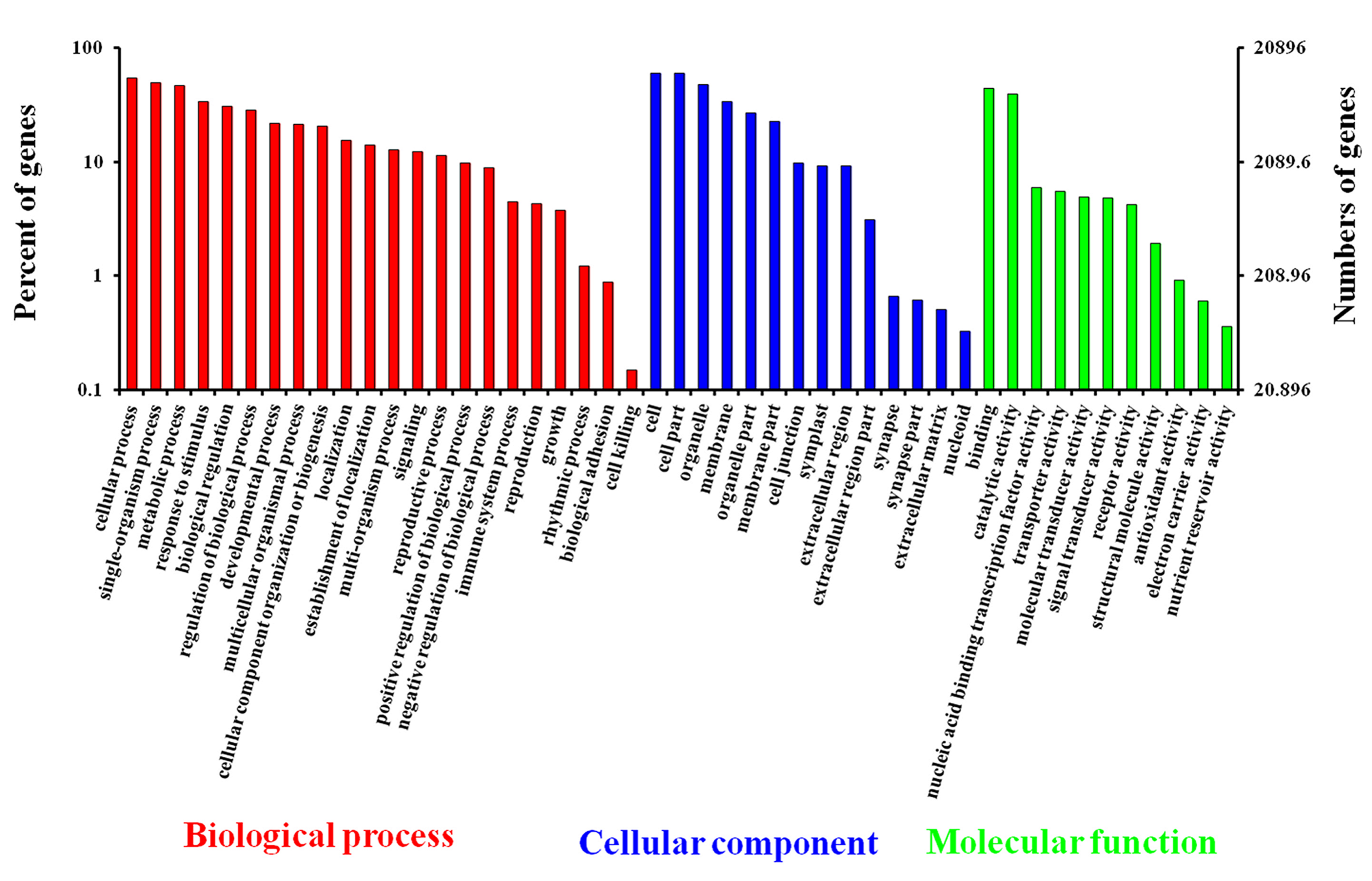

Supplement: S1 Fig — (TIF) [file pone.0229490.s001.tif]

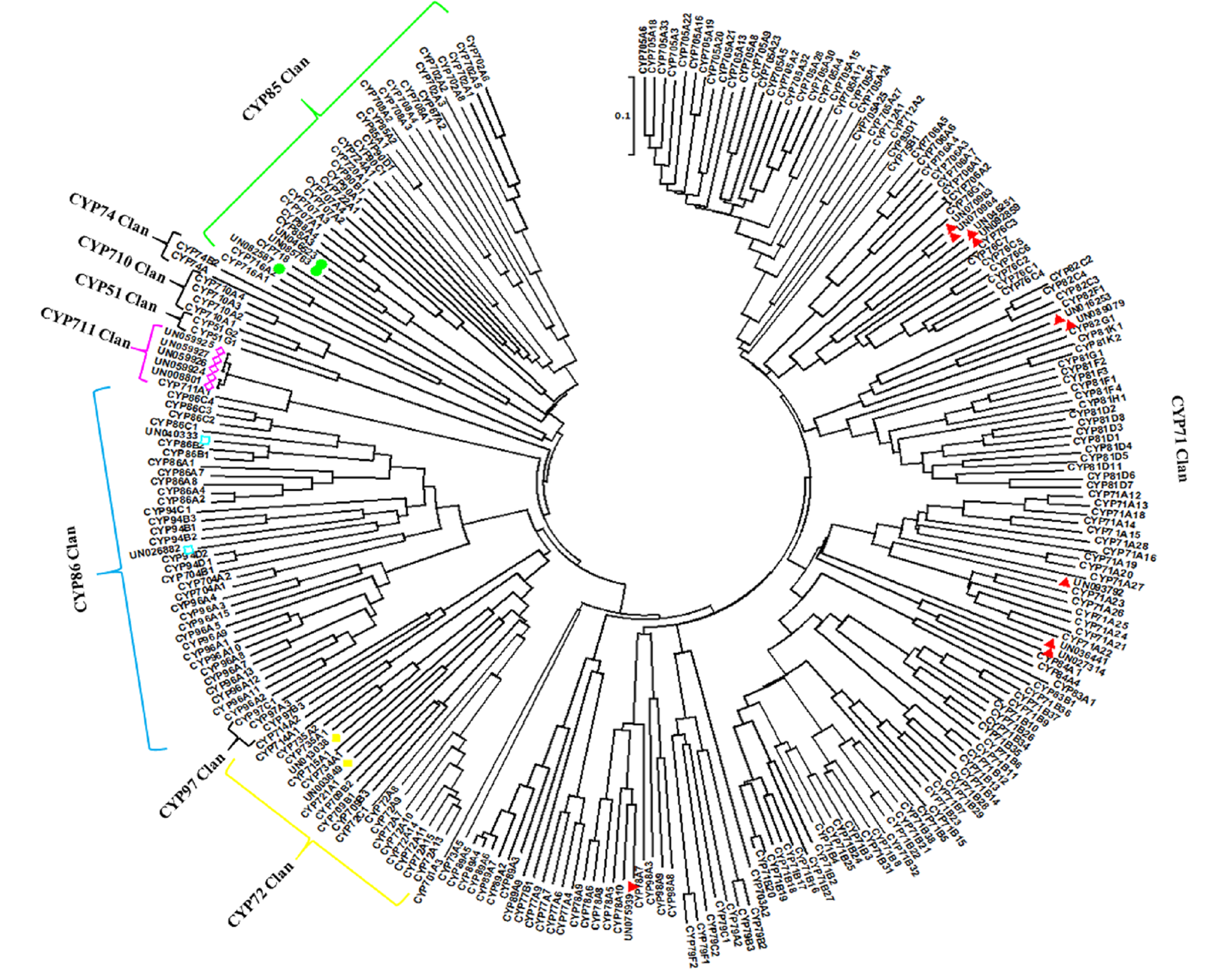

Supplement: S2 Fig — The phylogenetic tree was generated using the neighbor-joining (NJ) method in MEGA6. (TIF) [file pone.0229490.s002.tif]

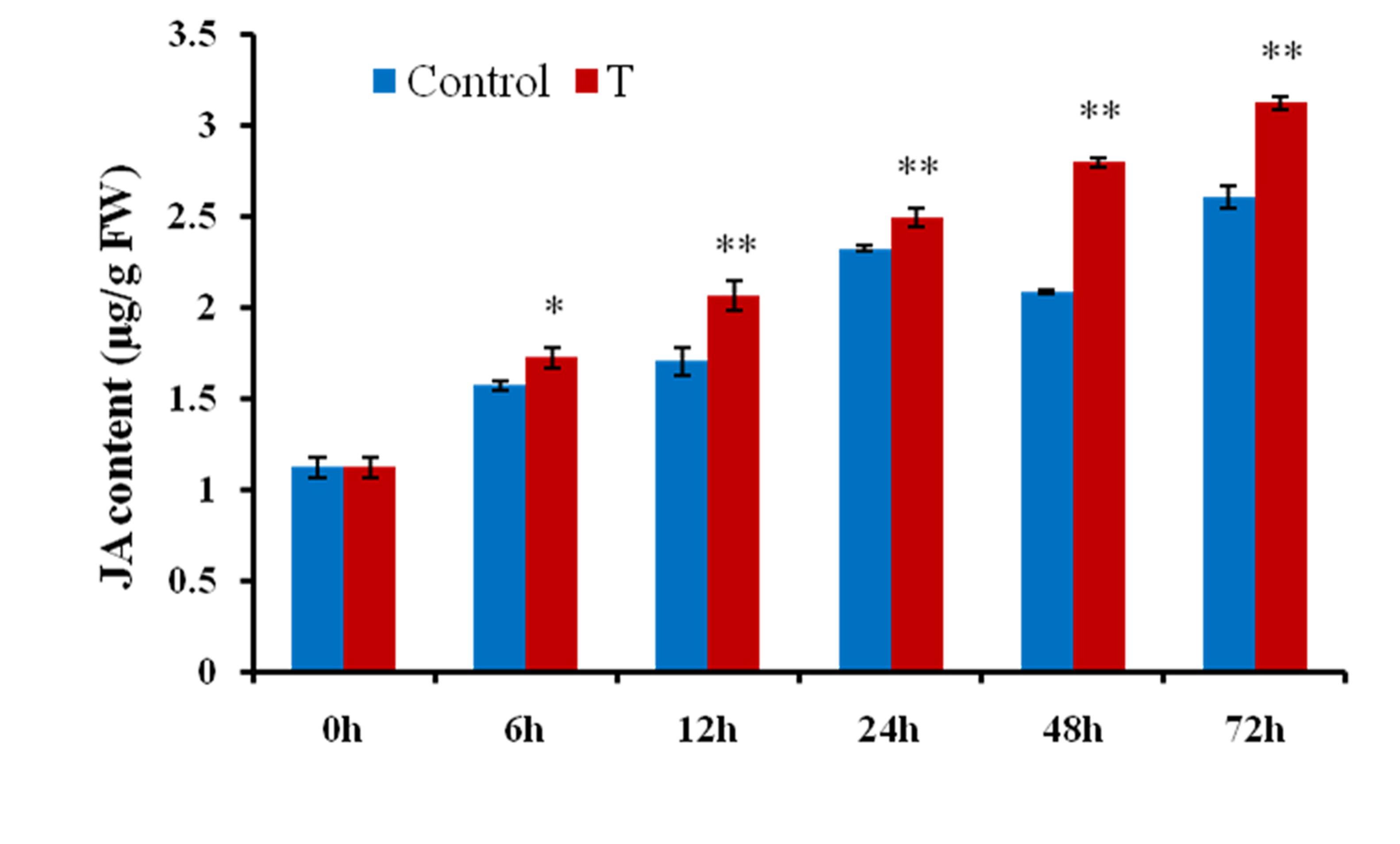

Supplement: S3 Fig — (TIF) [file pone.0229490.s003.tif]

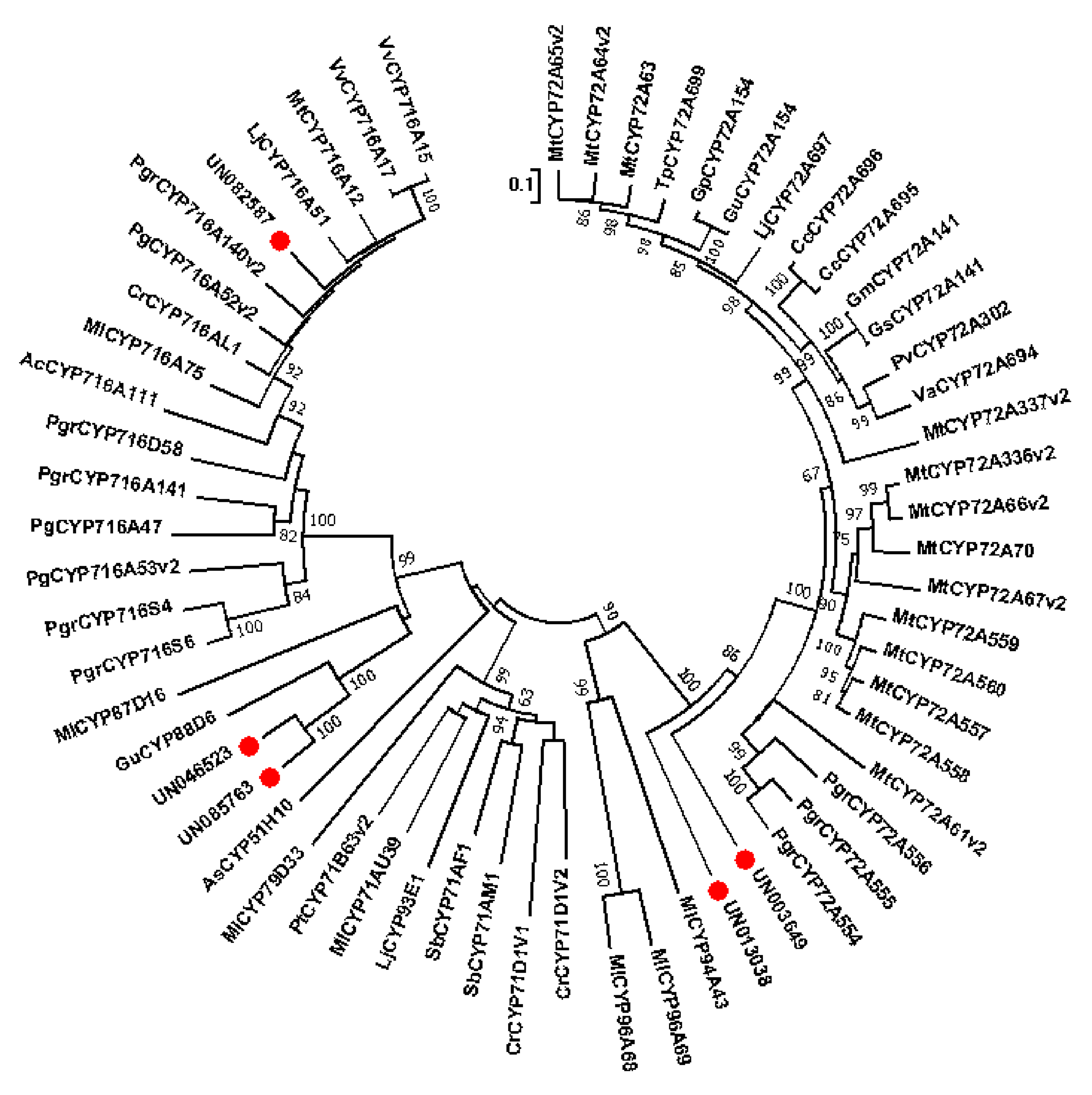

Supplement: S4 Fig — The phylogenetic tree was generated using the neighbor-joining (NJ) method in MEGA6. (TIF) [file pone.0229490.s004.tif]
