# Supplementary material for: NAA and 6-BA promote accumulation of oleanolic acid by JA regulation in Achyranthes bidentata Bl
Source: PLoS One. 2020 Feb 27;15(2):e0229490. doi: 10.1371/journal.pone.0229490 (PMC7046271; doi:10.1371/journal.pone.0229490)
Supplement: S1 Table — (DOCX) [file pone.0229490.s005.docx]

**Table S1. Primers used for qRT-PCR analysis.**

| **Accession No.** | **Annotation** | **Primer set** | |
| --- | --- | --- | --- |
|  |  | **Forward primer (5’-3’)** | **Reverse primer (5’-3’)** |
| UN045093 | *HMGR2* | TCTGTTGTTTGTGAGGCTGTT | GGCGATGTAGATGGCTGAC |
| UN046670 | *PMK3* | TGGGTTGGGTTCCTCTGC | CTTTCCCTTGGGCGATACA |
| UN041060 | *FPS7* | GATACTTCTCGTCAATGGGTT | CTTCTCCGTCCTTCAGCA |
| UN043163 | *SS2* | ATGGCGACAAATGCCTTAT | CACCACACCTCTGAACACTTG |
| UN045622 | *SE1* | GCAGGTGCTTTATACAAGGT | CAATGGTTTAGGGTTCAGG |
| Contig935 | *beta-AS1* | CCTCAAGTGTTGCCTAAT | GCCCTCCTTTCTTACTCT |
| UN011760 | *actin* | CCAAGGGCTGTCTTTCCA | TAGGCATCCTTCTGTCCCAT |
