# Supplementary material for: NAA and 6-BA promote accumulation of oleanolic acid by JA regulation in Achyranthes bidentata Bl
Source: PLoS One. 2020 Feb 27;15(2):e0229490. doi: 10.1371/journal.pone.0229490 (PMC7046271; doi:10.1371/journal.pone.0229490)
Supplement: S2 Table — (DOCX) [file pone.0229490.s006.docx]

**Table S2. Photosynthetic pigment content in leaves.**

| **The content** | **chlorophyll a (mg/g）** | **chlorophyll b (mg/g）** | **carotenoid (mg/g）** |
| --- | --- | --- | --- |
| Control | 1.658±0.075 | 0.356±0.03 | 0.356±0.013 |
| Treated | 1.214±0.035^**^ | 0.266±0.018^*^ | 0.268±0.012^**^ |

Data represent the mean value ± standard error of three independent experiments.
