# Supplementary material for: NAA and 6-BA promote accumulation of oleanolic acid by JA regulation in Achyranthes bidentata Bl
Source: PLoS One. 2020 Feb 27;15(2):e0229490. doi: 10.1371/journal.pone.0229490 (PMC7046271; doi:10.1371/journal.pone.0229490)
Supplement: S3 Table — (DOCX) [file pone.0229490.s007.docx]

**Table S3. List of transcription factors.**

| **ID** | **Description** | **L_CK vs L_T** | | | | | | | |  |
| --- | --- | --- | --- | --- | --- | --- | --- | --- | --- | --- |
|  |  | **L_CK_rep1** | **L_CK_rep2** | **mean** | **L_T_rep1** | **L_T_rep2** | **mean** | **ratio** | **adjust p** | |
| UN099018 | Myb transcription factor | 0.00 | 0.00 | 0.00 | 6.48 | 7.56 | 7.02 | 702.00 | 1.15E-23 | |
| UN059876 | Myb transcription factor | 0.00 | 0.00 | 0.00 | 0.97 | 1.87 | 1.42 | 142.00 | 1.17E-04 | |
| UN060563 | MYB transcription factor | 5.00 | 4.34 | 4.67 | 8.39 | 10.43 | 9.41 | 2.01 | 8.17E-03 | |
| UN031879 | MYB transcription factor | 6.05 | 8.47 | 7.26 | 1.87 | 2.56 | 2.21 | 0.31 | 1.56E-03 | |
| UN014368 | MYB-related transcription factor | 0.04 | 0.00 | 0.02 | 1.95 | 1.98 | 1.96 | 98.25 | 5.74E-08 | |
| UN019866 | Myb-related transcription factor | 0.91 | 1.03 | 0.97 | 3.41 | 3.77 | 3.59 | 3.70 | 4.20E-03 | |
| UN023074 | Myb-like transcription factor family protein | 1.01 | 0.73 | 0.87 | 2.66 | 4.59 | 3.62 | 4.17 | 2.13E-02 | |
| UN003741 | Myb-like transcription factor family protein | 2.03 | 1.86 | 1.94 | 5.01 | 7.59 | 6.30 | 3.24 | 4.69E-03 | |
| UN053686 | Myb-like transcription factor family protein | 1.33 | 1.00 | 1.17 | 3.41 | 2.92 | 3.17 | 2.72 | 1.16E-02 | |
| UN053683 | myb-like transcription factor family protein | 9.15 | 5.95 | 7.55 | 18.09 | 17.18 | 17.63 | 2.34 | 3.60E-04 | |
| UN053685 | myb-like transcription factor family protein | 6.36 | 5.11 | 5.74 | 13.21 | 12.53 | 12.87 | 2.24 | 2.37E-03 | |
| UN031880 | MYB family transcription factor | 37.08 | 28.29 | 32.69 | 19.15 | 11.87 | 15.51 | 0.47 | 2.97E-06 | |
| UN057993 | Myb family transcription factor family protein | 5.01 | 4.74 | 4.88 | 1.41 | 2.50 | 1.96 | 0.40 | 4.68E-04 | |
| UN005266 | Myb family transcription factor family protein | 1.90 | 1.39 | 1.65 | 0.74 | 0.50 | 0.62 | 0.38 | 1.50E-02 | |
| UN031878 | Myb family transcription factor family protein | 16.98 | 15.09 | 16.04 | 6.91 | 5.36 | 6.13 | 0.38 | 1.31E-09 | |
| UN053682 | myb-like transcription factor family protein | 17.69 | 16.14 | 16.91 | 6.57 | 5.63 | 6.10 | 0.36 | 2.76E-10 | |
| UN044505 | Myb family transcription factor family protein | 4.61 | 5.05 | 4.83 | 1.17 | 0.44 | 0.80 | 0.17 | 1.54E-09 | |
| UN056805 | Heat stress transcription factor A-6b-like protein | 0.02 | 0.00 | 0.01 | 5.59 | 5.54 | 5.56 | 556.50 | 5.43E-37 | |
| UN024072 | Heat stress transcription factor B-4 | 0.07 | 0.09 | 0.08 | 10.64 | 7.74 | 9.19 | 114.88 | 2.10E-14 | |
| UN013779 | Heat stress transcription factor B-2b-like protein | 0.00 | 0.00 | 0.00 | 0.86 | 1.18 | 1.02 | 102.00 | 4.46E-02 | |
| UN073520 | Heat stress transcription factor A-6b | 0.56 | 0.74 | 0.65 | 10.43 | 5.03 | 7.73 | 11.89 | 1.08E-02 | |
| UN073521 | Heat stress transcription factor A-6b | 0.56 | 0.73 | 0.65 | 9.61 | 5.28 | 7.45 | 11.54 | 1.78E-03 | |
| UN073519 | Heat stress transcription factor A-6b | 0.63 | 0.87 | 0.75 | 11.15 | 5.70 | 8.43 | 11.23 | 6.05E-03 | |
| UN073522 | Heat stress transcription factor A-6b | 0.46 | 0.73 | 0.59 | 8.55 | 4.37 | 6.46 | 10.86 | 6.96E-03 | |
| UN002503 | Heat stress transcription factor B-2b-like protein | 0.15 | 0.20 | 0.17 | 1.84 | 1.67 | 1.75 | 10.03 | 4.68E-06 | |
| UN036596 | Heat stress transcription factor B-4 | 1.34 | 1.41 | 1.38 | 7.66 | 5.80 | 6.73 | 4.89 | 1.66E-06 | |
| UN036595 | Heat stress transcription factor B-4 | 1.99 | 2.37 | 2.18 | 11.90 | 9.26 | 10.58 | 4.85 | 1.64E-07 | |
| UN056810 | Heat stress transcription factor A-1 | 3.21 | 2.21 | 2.71 | 5.67 | 5.53 | 5.60 | 2.07 | 8.01E-03 | |
| UN056811 | Heat stress transcription factor A-1 | 3.37 | 2.38 | 2.88 | 5.98 | 5.80 | 5.89 | 2.05 | 8.88E-03 | |
| UN063326 | Heat stress transcription factor C-1 | 10.13 | 13.99 | 12.06 | 5.40 | 6.68 | 6.04 | 0.50 | 2.38E-02 | |
| UN037500 | Heat stress transcription factor A-4a-like protein | 7.27 | 7.54 | 7.40 | 3.74 | 3.56 | 3.65 | 0.49 | 3.33E-05 | |
| UN036008 | Heat stress transcription factor A3 | 11.74 | 8.78 | 10.26 | 3.95 | 4.73 | 4.34 | 0.42 | 1.31E-11 | |
| UN036006 | Heat stress transcription factor A3 | 12.87 | 9.70 | 11.29 | 4.08 | 5.19 | 4.63 | 0.41 | 3.64E-12 | |
| UN036007 | Heat stress transcription factor A3 | 10.76 | 8.17 | 9.46 | 3.39 | 4.19 | 3.79 | 0.40 | 6.67E-11 | |
| UN018373 | Heat stress transcription factor B-2a | 14.99 | 17.70 | 16.34 | 4.35 | 3.95 | 4.15 | 0.25 | 6.30E-10 | |
| UN073517 | Heat stress transcription factor A-6b | 6.04 | 4.42 | 5.23 | 1.40 | 0.98 | 1.19 | 0.23 | 5.17E-05 | |
| UN044372 | MADS-box transcription factor 27 | 0.00 | 0.00 | 0.00 | 5.03 | 5.97 | 5.50 | 550.00 | 1.86E-29 | |
| UN044373 | MADS-box transcription factor 27 | 0.00 | 0.00 | 0.00 | 4.84 | 5.60 | 5.22 | 522.00 | 2.65E-26 | |
| UN044374 | MADS-box transcription factor 27 | 0.00 | 0.00 | 0.00 | 4.16 | 5.00 | 4.58 | 458.00 | 5.44E-25 | |
| UN044371 | MADS-box transcription factor 27 | 0.00 | 0.00 | 0.00 | 3.89 | 4.55 | 4.22 | 422.00 | 1.14E-21 | |
| UN086406 | MADS box transcription factor | 0.00 | 0.00 | 0.00 | 4.25 | 2.56 | 3.41 | 340.50 | 1.56E-07 | |
| UN069059 | MADS-box transcription factor 27 | 0.00 | 0.00 | 0.00 | 2.82 | 1.85 | 2.33 | 233.50 | 2.74E-04 | |
| UN037623 | MADS-box transcription factor 27 | 0.10 | 0.04 | 0.07 | 7.40 | 4.81 | 6.11 | 87.21 | 8.40E-10 | |
| UN037624 | MADS-box transcription factor 27 | 0.03 | 0.04 | 0.04 | 3.28 | 1.87 | 2.58 | 73.57 | 8.79E-06 | |
| UN037622 | MADS-box transcription factor 27 | 0.07 | 0.04 | 0.06 | 5.02 | 3.04 | 4.03 | 73.27 | 4.04E-07 | |
| UN037619 | MADS-box transcription factor 27 | 0.06 | 0.00 | 0.03 | 1.66 | 1.66 | 1.66 | 55.33 | 5.47E-09 | |
| UN037618 | MADS-box transcription factor | 0.15 | 0.09 | 0.12 | 6.08 | 3.91 | 5.00 | 41.62 | 1.60E-08 | |
| UN037616 | MADS-box transcription factor | 0.16 | 0.07 | 0.12 | 5.21 | 3.31 | 4.26 | 37.04 | 7.83E-08 | |
| UN037620 | MADS-box transcription factor 27 | 0.14 | 0.07 | 0.11 | 4.43 | 2.77 | 3.60 | 34.29 | 3.09E-07 | |
| UN037625 | MADS-box transcription factor | 0.18 | 0.05 | 0.11 | 4.34 | 3.11 | 3.72 | 32.39 | 7.80E-12 | |
| UN037617 | MADS-box transcription factor 27 | 0.16 | 0.05 | 0.11 | 3.75 | 2.70 | 3.23 | 30.71 | 1.02E-11 | |
| UN037041 | MADS-box transcription factor | 0.55 | 0.14 | 0.35 | 4.92 | 4.85 | 4.88 | 14.16 | 2.05E-05 | |
| UN033780 | MADS-box transcription factor | 0.10 | 0.25 | 0.17 | 1.94 | 2.22 | 2.08 | 11.89 | 3.07E-07 | |
| UN021308 | K-box region and MADS-box transcription factor family protein | 0.21 | 0.27 | 0.24 | 2.63 | 2.54 | 2.58 | 10.77 | 3.47E-03 | |
| UN033781 | MADS-box transcription factor 23 | 0.10 | 0.26 | 0.18 | 1.55 | 2.29 | 1.92 | 10.67 | 2.76E-06 | |
| UN033779 | MADS-box transcription factor 29 | 0.10 | 0.29 | 0.20 | 1.64 | 2.06 | 1.85 | 9.49 | 3.83E-06 | |
| UN033778 | MADS-box transcription factor 23 | 0.10 | 0.30 | 0.20 | 1.40 | 2.13 | 1.76 | 8.82 | 2.77E-05 | |
| UN043365 | MADS box transcription factor | 0.81 | 0.39 | 0.60 | 4.63 | 5.00 | 4.81 | 8.02 | 2.00E-04 | |
| UN037038 | MADS-box transcription factor | 1.12 | 1.10 | 1.11 | 7.12 | 7.48 | 7.30 | 6.58 | 2.89E-10 | |
| UN037039 | MADS-box transcription factor | 1.04 | 0.86 | 0.95 | 5.19 | 6.24 | 5.71 | 6.02 | 2.27E-08 | |
| UN086844 | Type I MADS-box transcription factor | 1.28 | 1.14 | 1.21 | 5.79 | 6.94 | 6.37 | 5.26 | 9.63E-08 | |
| UN043363 | MADS box transcription factor | 2.09 | 0.93 | 1.51 | 7.06 | 7.78 | 7.42 | 4.91 | 3.41E-09 | |
| UN043362 | MADS box transcription factor | 2.84 | 1.50 | 2.17 | 9.47 | 9.46 | 9.46 | 4.36 | 2.43E-08 | |
| UN037042 | MADS box transcription factor | 19.08 | 19.06 | 19.07 | 9.07 | 9.80 | 9.44 | 0.49 | 5.83E-09 | |
| UN037043 | MADS box transcription factor | 24.58 | 25.83 | 25.20 | 11.57 | 12.23 | 11.90 | 0.47 | 2.45E-10 | |
| UN018199 | MADS-box transcription factor | 1.13 | 1.33 | 1.23 | 0.22 | 0.47 | 0.34 | 0.28 | 1.35E-02 | |
| UN036550 | MADS-box transcription factor protein 1 | 2.72 | 2.75 | 2.73 | 0.61 | 0.63 | 0.62 | 0.23 | 4.59E-06 | |
| UN036553 | MADS-box transcription factor protein 1 | 2.79 | 2.75 | 2.77 | 0.59 | 0.67 | 0.63 | 0.23 | 3.19E-06 | |
| UN036551 | MADS-box transcription factor protein 2 | 2.26 | 2.17 | 2.21 | 0.41 | 0.57 | 0.49 | 0.22 | 1.20E-05 | |
| UN036555 | MADS-box transcription factor protein 2 | 2.33 | 2.18 | 2.25 | 0.40 | 0.60 | 0.50 | 0.22 | 8.27E-06 | |
| UN026444 | Ethylene-responsive transcription factor 1 | 0.00 | 0.00 | 0.00 | 4.54 | 3.59 | 4.06 | 406.50 | 2.12E-15 | |
| UN026443 | Ethylene-responsive transcription factor 1 | 0.00 | 0.00 | 0.00 | 3.23 | 1.88 | 2.55 | 255.50 | 4.42E-06 | |
| UN001900 | Ethylene-responsive transcription factor 10 | 0.53 | 0.32 | 0.43 | 25.66 | 23.72 | 24.69 | 58.09 | 6.49E-64 | |
| UN079028 | Ethylene-responsive transcription factor 10 | 0.53 | 0.32 | 0.43 | 23.70 | 22.63 | 23.16 | 54.51 | 1.83E-60 | |
| UN003862 | Ethylene-responsive transcription factor 1 | 0.61 | 0.63 | 0.62 | 17.62 | 29.87 | 23.75 | 38.30 | 7.11E-08 | |
| UN005504 | Ethylene-responsive transcription factor | 1.88 | 1.82 | 1.85 | 27.68 | 34.43 | 31.05 | 16.79 | 2.87E-33 | |
| UN035439 | Ethylene-responsive transcription factor | 1.53 | 1.67 | 1.60 | 19.18 | 23.24 | 21.21 | 13.26 | 7.95E-39 | |
| UN002297 | Ethylene responsive transcription factor 2b | 12.45 | 14.46 | 13.46 | 122.76 | 122.47 | 122.62 | 9.11 | 2.23E-45 | |
| UN035440 | Ethylene-responsive transcription factor | 1.92 | 2.17 | 2.04 | 14.28 | 16.65 | 15.46 | 7.56 | 4.28E-24 | |
| UN005505 | Ethylene-responsive transcription factor | 2.79 | 2.75 | 2.77 | 16.78 | 19.19 | 17.98 | 6.49 | 2.78E-19 | |
| UN046631 | Ethylene-responsive transcription factor 4 | 8.56 | 6.69 | 7.62 | 33.20 | 33.72 | 33.46 | 4.39 | 1.24E-17 | |
| UN046632 | Ethylene-responsive transcription factor 4 | 7.63 | 6.44 | 7.04 | 28.22 | 27.45 | 27.84 | 3.96 | 1.74E-14 | |
| UN046633 | Ethylene-responsive transcription factor 4 | 9.52 | 8.35 | 8.93 | 34.61 | 33.50 | 34.05 | 3.81 | 2.05E-14 | |
| UN046635 | Ethylene-responsive transcription factor | 9.30 | 8.08 | 8.69 | 28.66 | 33.52 | 31.09 | 3.58 | 1.30E-08 | |
| UN007132 | Ethylene-responsive transcription factor 1A | 2.17 | 2.34 | 2.25 | 7.55 | 7.62 | 7.58 | 3.36 | 2.67E-06 | |
| UN046636 | Ethylene-responsive transcription factor | 92.09 | 91.23 | 91.66 | 221.19 | 228.79 | 224.99 | 2.45 | 6.20E-07 | |
| UN064348 | Ethylene-responsive transcription factor 4 | 35.63 | 30.97 | 33.30 | 71.08 | 75.10 | 73.09 | 2.19 | 7.82E-05 | |
| UN046634 | Ethylene-responsive transcription factor, putative | 28.11 | 29.71 | 28.91 | 57.81 | 63.31 | 60.56 | 2.09 | 1.50E-04 | |
| UN066259 | Ethylene-responsive transcription factor, putative | 61.97 | 55.42 | 58.70 | 127.93 | 125.51 | 126.72 | 2.16 | 6.29E-05 | |
| UN002339 | Ethylene-responsive transcription factor | 33.00 | 34.82 | 33.91 | 70.82 | 70.53 | 70.67 | 2.08 | 1.29E-04 | |
| UN028195 | Ethylene-responsive transcription factor 2 | 58.73 | 69.94 | 64.33 | 27.17 | 32.22 | 29.70 | 0.46 | 3.77E-05 | |
| UN051692 | Ethylene-responsive transcription factor 2 | 9.55 | 7.60 | 8.57 | 4.96 | 2.56 | 3.76 | 0.44 | 4.49E-03 | |
| UN076749 | Ethylene-responsive transcription factor 2 | 95.18 | 114.69 | 104.94 | 39.05 | 40.73 | 39.89 | 0.38 | 3.71E-06 | |
| UN100008 | Ethylene-responsive transcription factor, putative | 3.09 | 2.99 | 3.04 | 0.73 | 1.47 | 1.10 | 0.36 | 1.47E-03 | |
| UN086680 | Ethylene-responsive transcription factor, putative | 7.83 | 10.05 | 8.94 | 3.05 | 2.94 | 3.00 | 0.34 | 7.82E-05 | |
| UN099976 | Ethylene-responsive transcription factor 1B | 4.99 | 6.54 | 5.77 | 2.56 | 1.38 | 1.97 | 0.34 | 1.13E-03 | |
| UN031116 | Ethylene-responsive transcription factor 1B, putative | 14.82 | 13.93 | 14.38 | 3.88 | 4.01 | 3.94 | 0.27 | 9.20E-16 | |
| UN031117 | Ethylene-responsive transcription factor 1B, putative | 14.38 | 13.12 | 13.75 | 3.48 | 3.75 | 3.62 | 0.26 | 3.47E-17 | |
| UN031850 | Ethylene-responsive transcription factor ERF061 | 9.01 | 11.18 | 10.09 | 2.51 | 2.29 | 2.40 | 0.24 | 3.00E-07 | |
| UN077640 | Ethylene-responsive transcription factor, putative | 6.43 | 4.50 | 5.46 | 1.55 | 0.98 | 1.27 | 0.23 | 1.15E-10 | |
| UN099427 | Ethylene-responsive transcription factor 12 | 1.42 | 1.34 | 1.38 | 0.21 | 0.00 | 0.10 | 0.08 | 1.04E-05 | |
| UN065005 | AP2-like ethylene-responsive transcription factor | 0.73 | 0.55 | 0.64 | 11.03 | 9.40 | 10.21 | 15.96 | 2.86E-32 | |
| UN040605 | AP2-like ethylene-responsive transcription factor | 0.35 | 0.24 | 0.29 | 3.32 | 5.69 | 4.50 | 15.27 | 4.43E-05 | |
| UN028073 | AP2-like ethylene-responsive transcription factor | 2.28 | 2.08 | 2.18 | 30.13 | 33.57 | 31.85 | 14.61 | 4.78E-55 | |
| UN065006 | AP2-like ethylene-responsive transcription factor | 1.20 | 0.65 | 0.93 | 14.38 | 12.59 | 13.48 | 14.58 | 2.26E-39 | |
| UN006104 | AP2-like ethylene-responsive transcription factor | 0.31 | 0.20 | 0.26 | 2.95 | 4.47 | 3.71 | 14.55 | 1.00E-07 | |
| UN028072 | AP2-like ethylene-responsive transcription factor | 1.97 | 1.67 | 1.82 | 23.81 | 27.04 | 25.42 | 13.97 | 3.27E-52 | |
| UN065004 | AP2-like ethylene-responsive transcription factor | 1.82 | 1.00 | 1.41 | 17.20 | 15.14 | 16.17 | 11.47 | 1.49E-36 | |
| UN065003 | AP2-like ethylene-responsive transcription factor | 1.38 | 0.84 | 1.11 | 13.43 | 11.78 | 12.61 | 11.36 | 2.17E-35 | |
| UN048191 | AP2-like ethylene-responsive transcription factor | 0.85 | 0.77 | 0.81 | 6.95 | 7.09 | 7.02 | 8.67 | 1.40E-16 | |
| UN048192 | AP2-like ethylene-responsive transcription factor | 0.29 | 0.37 | 0.33 | 2.42 | 3.12 | 2.77 | 8.39 | 2.58E-06 | |
| UN006911 | AP2-like ethylene-responsive transcription factor | 0.58 | 0.46 | 0.52 | 3.89 | 4.21 | 4.05 | 7.79 | 5.23E-13 | |
| UN048189 | AP2-like ethylene-responsive transcription factor | 0.96 | 0.98 | 0.97 | 4.47 | 4.36 | 4.42 | 4.55 | 1.19E-07 | |
| UN048190 | AP2-like ethylene-responsive transcription factor | 1.40 | 1.18 | 1.29 | 5.71 | 5.66 | 5.69 | 4.41 | 1.29E-07 | |
| UN003381 | AP2-like ethylene-responsive transcription factor | 0.66 | 0.54 | 0.60 | 2.64 | 2.64 | 2.64 | 4.40 | 1.55E-04 | |
| UN059247 | AP2-like ethylene-responsive transcription factor | 2.83 | 2.97 | 2.90 | 10.72 | 11.64 | 11.18 | 3.86 | 3.32E-12 | |
| UN059242 | AP2-like ethylene-responsive transcription factor | 3.16 | 3.28 | 3.22 | 11.96 | 12.66 | 12.31 | 3.82 | 4.05E-12 | |
| UN059246 | AP2-like ethylene-responsive transcription factor | 2.50 | 2.64 | 2.57 | 9.06 | 10.29 | 9.68 | 3.76 | 1.45E-11 | |
| UN059245 | AP2-like ethylene-responsive transcription factor | 2.64 | 2.16 | 2.40 | 7.74 | 8.74 | 8.24 | 3.43 | 6.89E-07 | |
| UN059250 | AP2-like ethylene-responsive transcription factor | 4.36 | 4.66 | 4.51 | 10.40 | 10.15 | 10.28 | 2.28 | 2.90E-04 | |
| UN015160 | AP2-like ethylene-responsive transcription factor | 1.93 | 2.09 | 2.01 | 0.70 | 0.63 | 0.67 | 0.33 | 1.61E-03 | |
| UN015161 | AP2-like ethylene-responsive transcription factor | 1.98 | 2.40 | 2.19 | 0.51 | 0.62 | 0.56 | 0.26 | 6.60E-05 | |
| UN099208 | WRKY transcription factor 6 | 0.00 | 0.00 | 0.00 | 4.14 | 3.33 | 3.73 | 373.50 | 3.88E-20 | |
| UN068520 | WRKY transcription factor 6 | 0.00 | 0.00 | 0.00 | 1.56 | 1.20 | 1.38 | 138.00 | 3.43E-02 | |
| UN022634 | WRKY transcription factor 18 | 24.83 | 11.30 | 18.06 | 57.19 | 63.57 | 60.38 | 3.34 | 6.39E-06 | |
| UN011521 | WRKY transcription factor, putative | 27.96 | 30.64 | 29.30 | 61.88 | 70.65 | 66.27 | 2.26 | 9.92E-06 | |
| UN030929 | WRKY transcription factor 40 | 19.50 | 24.53 | 22.02 | 13.75 | 8.15 | 10.95 | 0.50 | 9.89E-03 | |
| UN032074 | WRKY 7 transcription factor | 23.68 | 20.27 | 21.98 | 10.58 | 11.51 | 11.04 | 0.50 | 6.79E-05 | |
| UN049945 | WRKY family transcription factor | 6.34 | 5.96 | 6.15 | 2.88 | 2.95 | 2.92 | 0.47 | 1.20E-05 | |
| UN052407 | WRKY family transcription factor | 8.03 | 4.57 | 6.30 | 2.46 | 3.24 | 2.85 | 0.45 | 1.58E-02 | |
| UN052421 | WRKY transcription factor, putative | 17.30 | 11.67 | 14.48 | 5.70 | 6.84 | 6.27 | 0.43 | 2.08E-05 | |
| UN015732 | WRKY transcription factor-a | 2.90 | 2.53 | 2.71 | 0.75 | 1.16 | 0.95 | 0.35 | 4.11E-03 | |
| UN011486 | WRKY transcription factor-a | 3.29 | 2.06 | 2.67 | 0.68 | 1.16 | 0.92 | 0.34 | 3.81E-04 | |
| UN068276 | WRKY transcription factor, putative | 3.32 | 3.05 | 3.18 | 1.38 | 0.66 | 1.02 | 0.32 | 2.20E-04 | |
| UN051857 | WRKY transcription factor 2-4 | 1.17 | 1.46 | 1.31 | 0.24 | 0.56 | 0.40 | 0.30 | 8.62E-03 | |
| UN023128 | WRKY transcription factor 12 | 1.16 | 1.26 | 1.21 | 0.30 | 0.19 | 0.24 | 0.20 | 4.55E-02 | |
| UN013554 | Transcription factor bHLH25 | 0.93 | 0.93 | 0.93 | 284.62 | 230.49 | 257.56 | 276.94 | 2.24E-54 | |
| UN094694 | Basic helix-loop-helix transcription factor | 0.00 | 0.00 | 0.00 | 1.22 | 0.64 | 0.93 | 93.00 | 4.33E-02 | |
| UN099492 | Basic helix loop helix (bHLH) family transcription factor | 0.07 | 0.00 | 0.04 | 1.09 | 0.52 | 0.81 | 23.00 | 1.35E-02 | |
| UN018253 | Basic helix loop helix (bHLH) family transcription factor | 0.03 | 0.18 | 0.10 | 2.01 | 1.67 | 1.84 | 17.52 | 2.94E-07 | |
| UN050791 | Transcription factor bHLH62-like protein | 1.91 | 1.36 | 1.64 | 16.56 | 15.59 | 16.07 | 9.83 | 1.46E-17 | |
| UN011618 | Transcription factor bHLH93-like protein | 0.70 | 0.51 | 0.60 | 5.35 | 6.05 | 5.70 | 9.42 | 6.90E-11 | |
| UN051242 | Basic helix-loop-helix (bHLH) family transcription factor | 2.05 | 2.14 | 2.09 | 10.97 | 8.30 | 9.64 | 4.60 | 2.72E-06 | |
| UN051243 | Basic helix-loop-helix (bHLH) family transcription factor | 1.56 | 2.01 | 1.78 | 9.32 | 6.75 | 8.04 | 4.50 | 8.88E-05 | |
| UN051245 | Basic helix-loop-helix (bHLH) family transcription factor | 1.52 | 1.75 | 1.64 | 7.81 | 5.71 | 6.76 | 4.13 | 9.99E-05 | |
| UN047895 | bHLH transcription factor-like protein | 7.67 | 5.06 | 6.37 | 23.59 | 23.20 | 23.39 | 3.68 | 1.61E-11 | |
| UN000100 | bHLH transcription factor-like protein | 22.64 | 18.78 | 20.71 | 74.86 | 68.61 | 71.73 | 3.46 | 3.03E-14 | |
| UN047898 | bHLH transcription factor-like protein | 10.05 | 7.28 | 8.67 | 27.20 | 30.67 | 28.94 | 3.34 | 5.32E-11 | |
| UN011335 | bHLH transcription factor | 15.70 | 10.41 | 13.05 | 44.15 | 42.48 | 43.31 | 3.32 | 2.41E-12 | |
| UN045473 | bHLH transcription factor | 7.78 | 6.85 | 7.31 | 15.25 | 18.11 | 16.68 | 2.28 | 8.53E-05 | |
| UN045474 | bHLH transcription factor | 7.88 | 6.87 | 7.38 | 14.55 | 17.39 | 15.97 | 2.17 | 3.98E-04 | |
| UN045476 | bHLH transcription factor | 9.41 | 7.74 | 8.57 | 16.16 | 18.68 | 17.42 | 2.03 | 1.90E-03 | |
| UN051244 | Basic helix-loop-helix (bHLH) family transcription factor | 8.55 | 9.91 | 9.23 | 4.60 | 3.95 | 4.28 | 0.46 | 5.38E-06 | |
| UN070400 | Basic helix loop helix (bHLH) family transcription factor | 21.14 | 36.15 | 28.64 | 9.87 | 7.87 | 8.87 | 0.31 | 4.35E-02 | |
| UN052402 | Basic helix-loop-helix transcription factor | 6.13 | 8.03 | 7.08 | 2.13 | 1.54 | 1.83 | 0.26 | 2.76E-05 | |
| UN003341 | Transcription factor bHLH25-like protein | 40.56 | 62.07 | 51.31 | 14.23 | 5.22 | 9.72 | 0.19 | 1.51E-03 | |
| UN019647 | Transcription factor bHLH25-like protein | 28.72 | 45.95 | 37.34 | 9.92 | 3.65 | 6.79 | 0.18 | 3.10E-03 | |
| UN083783 | Transcription factor | 0.00 | 0.00 | 0.00 | 0.99 | 0.77 | 0.88 | 88.00 | 2.15E-04 | |
| UN026409 | Transcription factor | 0.15 | 0.12 | 0.14 | 1.43 | 0.72 | 1.07 | 7.96 | 3.37E-02 | |
| UN072200 | Transcription factor | 4.06 | 2.90 | 3.48 | 24.06 | 26.18 | 25.12 | 7.22 | 4.20E-33 | |
| UN026410 | Transcription factor | 0.16 | 0.12 | 0.14 | 1.20 | 0.68 | 0.94 | 6.71 | 1.64E-02 | |
| UN072199 | Transcription factor | 4.58 | 3.42 | 4.00 | 24.97 | 27.26 | 26.12 | 6.53 | 4.18E-30 | |
| UN072206 | Transcription factor | 4.83 | 3.68 | 4.25 | 25.22 | 27.87 | 26.55 | 6.24 | 1.18E-28 | |
| UN072202 | Transcription factor | 4.65 | 3.49 | 4.07 | 23.75 | 26.41 | 25.08 | 6.16 | 3.79E-27 | |
| UN072213 | Transcription factor | 4.29 | 3.27 | 3.78 | 22.14 | 23.26 | 22.70 | 6.01 | 1.00E-26 | |
| UN072207 | Transcription factor | 4.68 | 3.70 | 4.19 | 21.07 | 22.10 | 21.59 | 5.15 | 2.43E-21 | |
| UN054243 | Transcription factor | 0.74 | 0.96 | 0.85 | 2.96 | 2.91 | 2.94 | 3.45 | 2.15E-02 | |
| UN052808 | Transcription factor | 7.70 | 6.06 | 6.88 | 23.27 | 22.24 | 22.75 | 3.31 | 1.46E-09 | |
| UN052810 | Transcription factor | 8.97 | 7.30 | 8.13 | 25.76 | 25.39 | 25.58 | 3.14 | 6.67E-10 | |
| UN052812 | Transcription factor | 9.87 | 8.54 | 9.20 | 22.92 | 23.58 | 23.25 | 2.53 | 3.71E-06 | |
| UN054249 | Transcription factor | 1.33 | 1.09 | 1.21 | 3.14 | 2.80 | 2.97 | 2.45 | 1.31E-03 | |
| UN058956 | Transcription factor | 2.21 | 2.69 | 2.45 | 5.55 | 5.52 | 5.54 | 2.26 | 2.24E-02 | |
| UN054247 | Transcription factor | 1.97 | 1.46 | 1.71 | 3.99 | 3.74 | 3.87 | 2.25 | 5.39E-03 | |
| UN045475 | Transcription factor | 11.20 | 9.70 | 10.45 | 21.16 | 25.71 | 23.44 | 2.24 | 1.11E-04 | |
| UN054246 | Transcription factor | 1.87 | 1.57 | 1.72 | 3.94 | 3.68 | 3.81 | 2.22 | 1.71E-02 | |
| UN054244 | Transcription factor | 4.11 | 4.00 | 4.05 | 9.13 | 8.67 | 8.90 | 2.19 | 3.98E-03 | |
| UN045477 | Transcription factor | 11.33 | 9.73 | 10.53 | 20.30 | 24.82 | 22.56 | 2.14 | 4.37E-04 | |
| UN054248 | Transcription factor | 4.89 | 4.66 | 4.78 | 10.49 | 9.27 | 9.88 | 2.07 | 9.92E-03 | |
| UN054245 | Transcription factor | 3.20 | 2.35 | 2.78 | 5.78 | 5.62 | 5.70 | 2.05 | 3.90E-02 | |
| UN045478 | Transcription factor | 13.10 | 10.70 | 11.90 | 22.07 | 26.14 | 24.11 | 2.03 | 1.78E-03 | |
| UN033096 | Transcription factor | 8.05 | 9.34 | 8.70 | 3.21 | 4.30 | 3.75 | 0.43 | 4.29E-06 | |
| UN045686 | Transcription factor | 11.51 | 9.08 | 10.29 | 3.37 | 3.28 | 3.33 | 0.32 | 2.30E-14 | |
| UN045687 | Transcription factor | 17.41 | 14.84 | 16.12 | 4.33 | 4.83 | 4.58 | 0.28 | 2.88E-22 | |
| UN050786 | Transcription factor, putative | 1.17 | 1.34 | 1.25 | 26.07 | 23.47 | 24.77 | 19.74 | 7.95E-59 | |
| UN050787 | Transcription factor, putative | 3.13 | 2.75 | 2.94 | 27.97 | 26.04 | 27.00 | 9.19 | 2.06E-37 | |
| UN010729 | Transcription factor, putative | 25.07 | 18.70 | 21.88 | 129.36 | 147.24 | 138.30 | 6.32 | 1.72E-33 | |
| UN056508 | Transcription factor, putative | 24.54 | 21.16 | 22.85 | 120.93 | 128.51 | 124.72 | 5.46 | 2.83E-28 | |
| UN056510 | Transcription factor, putative | 24.39 | 21.18 | 22.79 | 120.33 | 127.10 | 123.72 | 5.43 | 2.37E-28 | |
| UN039824 | Transcription factor, putative | 0.92 | 1.10 | 1.01 | 3.50 | 3.44 | 3.47 | 3.44 | 1.52E-02 | |
| UN007976 | Transcription factor, putative | 27.21 | 17.96 | 22.59 | 74.53 | 73.51 | 74.02 | 3.28 | 5.31E-12 | |
| UN062651 | Transcription factor, putative | 1.28 | 0.90 | 1.09 | 3.77 | 3.00 | 3.38 | 3.11 | 7.01E-05 | |
| UN062648 | Transcription factor, putative | 1.47 | 1.04 | 1.25 | 3.85 | 3.43 | 3.64 | 2.90 | 9.82E-05 | |
| UN039826 | Transcription factor, putative | 1.31 | 1.13 | 1.22 | 3.24 | 3.63 | 3.44 | 2.82 | 9.68E-03 | |
| UN039827 | Transcription factor, putative | 1.78 | 2.03 | 1.90 | 4.71 | 4.73 | 4.72 | 2.48 | 8.68E-03 | |
| UN062646 | Transcription factor, putative | 2.00 | 1.11 | 1.56 | 3.81 | 3.26 | 3.54 | 2.27 | 2.68E-02 | |
| UN062647 | Transcription factor, putative | 2.28 | 1.34 | 1.81 | 4.27 | 3.58 | 3.92 | 2.17 | 3.83E-02 | |
| UN067736 | Transcription factor, putative | 11.25 | 13.00 | 12.12 | 5.31 | 5.66 | 5.48 | 0.45 | 1.30E-06 | |
| UN049041 | Transcription factor, putative | 4.64 | 3.94 | 4.29 | 1.68 | 2.07 | 1.88 | 0.44 | 4.37E-06 | |
| UN049046 | Transcription factor, putative | 4.53 | 3.70 | 4.12 | 1.61 | 2.00 | 1.80 | 0.44 | 5.67E-06 | |
| UN049049 | Transcription factor, putative | 4.61 | 3.84 | 4.22 | 1.49 | 1.92 | 1.71 | 0.40 | 5.47E-07 | |
| UN049050 | Transcription factor, putative | 4.51 | 3.62 | 4.06 | 1.42 | 1.85 | 1.64 | 0.40 | 6.93E-07 | |
| UN049039 | Transcription factor, putative | 2.92 | 2.70 | 2.81 | 0.74 | 1.38 | 1.06 | 0.38 | 1.07E-04 | |
| UN024580 | Transcription factor, putative | 2.46 | 3.93 | 3.20 | 1.31 | 0.53 | 0.92 | 0.29 | 3.12E-02 | |
| UN011368 | Transcription factor, putative | 161.77 | 106.92 | 134.34 | 17.52 | 19.75 | 18.63 | 0.14 | 1.11E-11 | |
| UN004081 | Transcription factor radialis-like protein | 0.00 | 0.00 | 0.00 | 2.58 | 1.38 | 1.98 | 198.00 | 2.71E-03 | |
| UN011541 | Transcription factor protein | 0.00 | 0.00 | 0.00 | 1.70 | 1.93 | 1.81 | 181.50 | 7.38E-11 | |
| UN014049 | Transcription factor protein | 0.67 | 0.68 | 0.68 | 8.55 | 6.89 | 7.72 | 11.44 | 1.24E-16 | |
| UN058466 | Transcription factor protein | 12.42 | 10.37 | 11.39 | 26.66 | 34.15 | 30.41 | 2.67 | 8.74E-06 | |
| UN041898 | Transcription factor LIM | 8.93 | 6.98 | 7.96 | 28.17 | 25.96 | 27.07 | 3.40 | 1.92E-10 | |
| UN006239 | Transcription factor LIM | 4.12 | 3.52 | 3.82 | 10.04 | 11.64 | 10.84 | 2.84 | 3.65E-05 | |
| UN011669 | Transcription factor ABORTED MICROSPORES | 1.23 | 0.88 | 1.05 | 3.19 | 2.58 | 2.88 | 2.73 | 4.90E-02 | |
| UN045480 | Transcription factor UNE12 | 5.90 | 4.12 | 5.01 | 11.84 | 12.85 | 12.34 | 2.46 | 1.60E-02 | |
| UN047068 | Transcription factor ILR3 | 2.38 | 2.47 | 2.42 | 5.25 | 5.96 | 5.61 | 2.31 | 2.78E-02 | |
| UN063942 | Transcription factor DP | 2.67 | 2.16 | 2.42 | 5.27 | 4.78 | 5.03 | 2.08 | 2.75E-02 | |
| UN079126 | transcription factor-related | 0.30 | 0.06 | 0.18 | 2.43 | 2.52 | 2.48 | 13.75 | 9.84E-07 | |
| UN020046 | Transcription factor-related, putative | 2.39 | 1.90 | 2.15 | 14.73 | 19.26 | 17.00 | 7.92 | 2.96E-14 | |
| UN009331 | Transcription factor-related family protein | 3.36 | 2.65 | 3.00 | 6.47 | 5.93 | 6.20 | 2.06 | 6.37E-03 | |
| UN098143 | Trihelix transcription factor GT-2 | 0.37 | 0.29 | 0.33 | 14.50 | 11.91 | 13.21 | 40.02 | 4.64E-32 | |
| UN005541 | Trihelix transcription factor GT-3a | 16.43 | 12.68 | 14.55 | 108.33 | 117.29 | 112.81 | 7.75 | 3.54E-40 | |
| UN078067 | Scarecrow transcription factor family protein | 0.64 | 0.85 | 0.74 | 9.08 | 10.89 | 9.98 | 13.40 | 1.21E-35 | |
| UN057305 | Scarecrow transcription factor family protein | 1.32 | 0.80 | 1.06 | 14.44 | 13.40 | 13.92 | 13.13 | 4.79E-42 | |
| UN057307 | Scarecrow transcription factor family protein | 1.32 | 0.82 | 1.07 | 14.21 | 13.16 | 13.69 | 12.79 | 1.41E-41 | |
| UN057309 | Scarecrow transcription factor family protein | 1.38 | 0.86 | 1.12 | 14.81 | 13.68 | 14.25 | 12.72 | 3.64E-42 | |
| UN057308 | Scarecrow transcription factor family protein | 1.38 | 0.88 | 1.13 | 14.57 | 13.44 | 14.00 | 12.39 | 1.00E-41 | |
| UN057306 | Scarecrow transcription factor family protein | 1.12 | 0.73 | 0.93 | 10.54 | 11.26 | 10.90 | 11.78 | 8.23E-37 | |
| UN008332 | Scarecrow transcription factor family protein | 1.12 | 0.76 | 0.94 | 10.44 | 11.10 | 10.77 | 11.46 | 2.00E-36 | |
| UN089061 | Scarecrow transcription factor family protein | 2.55 | 2.17 | 2.36 | 10.14 | 10.06 | 10.10 | 4.28 | 1.40E-10 | |
| UN001787 | Scarecrow-like 1 transcription factor | 2.28 | 1.22 | 1.75 | 0.77 | 0.90 | 0.83 | 0.48 | 4.78E-02 | |
| UN079505 | Scarecrow transcription factor family protein | 29.32 | 29.28 | 29.30 | 8.85 | 8.31 | 8.58 | 0.29 | 1.46E-24 | |
| UN004592 | bZIP transcription factor family protein | 0.88 | 1.02 | 0.95 | 11.20 | 13.86 | 12.53 | 13.19 | 1.50E-22 | |
| UN007163 | bZIP family transcription factor family protein | 7.22 | 6.96 | 7.09 | 3.98 | 3.18 | 3.58 | 0.50 | 1.60E-03 | |
| UN098464 | bZIP transcription factor FD-like protein | 25.47 | 29.76 | 27.62 | 3.88 | 4.21 | 4.04 | 0.15 | 2.16E-15 | |
| UN098463 | bZIP transcription factor FD-like protein | 34.94 | 37.12 | 36.03 | 5.08 | 5.04 | 5.06 | 0.14 | 9.71E-38 | |
| UN000257 | bZIP transcription factor | 6.66 | 7.41 | 7.04 | 2.59 | 3.48 | 3.04 | 0.43 | 4.99E-04 | |
| UN031604 | bZIP family transcription factor | 69.38 | 70.55 | 69.97 | 25.49 | 30.11 | 27.80 | 0.40 | 6.69E-15 | |
| UN031605 | bZIP family transcription factor | 48.64 | 46.39 | 47.52 | 17.63 | 19.06 | 18.34 | 0.39 | 1.61E-14 | |
| UN030730 | Basic-leucine zipper (bZIP) transcription factor family protein | 0.25 | 0.13 | 0.19 | 2.82 | 1.85 | 2.33 | 12.29 | 6.94E-05 | |
| UN012249 | Basic-leucine zipper (bZIP) transcription factor family protein | 1.87 | 1.78 | 1.83 | 12.62 | 6.95 | 9.79 | 5.36 | 2.46E-02 | |
| UN008191 | Basic-leucine zipper (bZIP) transcription factor family protein | 3.72 | 4.13 | 3.92 | 13.90 | 11.00 | 12.45 | 3.17 | 2.30E-05 | |
| UN048986 | Basic-leucine zipper (bZIP) transcription factor family protein | 7.02 | 4.11 | 5.56 | 17.39 | 16.38 | 16.88 | 3.03 | 2.49E-08 | |
| UN048987 | Basic-leucine zipper (bZIP) transcription factor family protein | 3.19 | 2.07 | 2.63 | 7.07 | 7.84 | 7.46 | 2.83 | 1.15E-05 | |
| UN056491 | Basic-leucine zipper (bZIP) transcription factor family protein | 4.23 | 3.65 | 3.94 | 11.10 | 8.79 | 9.95 | 2.52 | 5.60E-04 | |
| UN030729 | Basic-leucine zipper (bZIP) transcription factor family protein | 4.63 | 5.45 | 5.04 | 1.87 | 2.56 | 2.21 | 0.44 | 2.54E-04 | |
| UN031557 | Basic-leucine zipper (bZIP) transcription factor family protein | 9.33 | 11.28 | 10.30 | 5.16 | 3.41 | 4.29 | 0.42 | 1.72E-04 | |
| UN076153 | Basic-leucine zipper (bZIP) transcription factor family protein | 16.23 | 15.99 | 16.11 | 6.08 | 6.44 | 6.26 | 0.39 | 7.29E-12 | |
| UN031558 | Basic-leucine zipper (bZIP) transcription factor family protein | 3.70 | 4.34 | 4.02 | 1.42 | 1.60 | 1.51 | 0.38 | 3.80E-05 | |
| UN032137 | Basic-leucine zipper (bZIP) transcription factor family protein | 4.60 | 3.17 | 3.88 | 0.91 | 1.75 | 1.33 | 0.34 | 3.97E-05 | |
| UN032138 | Basic-leucine zipper (bZIP) transcription factor family protein | 4.70 | 3.27 | 3.99 | 0.98 | 1.61 | 1.29 | 0.32 | 1.72E-05 | |
| UN031560 | Basic-leucine zipper (bZIP) transcription factor family protein, putative | 8.17 | 8.30 | 8.23 | 2.64 | 3.28 | 2.96 | 0.36 | 1.37E-07 | |
| UN046748 | Basic-leucine zipper (bZIP) transcription factor family protein, putative | 7.14 | 4.78 | 5.96 | 0.99 | 0.68 | 0.83 | 0.14 | 6.09E-11 | |
| UN031602 | Transcription factor HY5, putative | 2.82 | 3.02 | 2.92 | 1.08 | 1.46 | 1.27 | 0.43 | 4.93E-02 | |
| UN031606 | Transcription factor HY5, putative | 3.57 | 2.83 | 3.20 | 0.64 | 1.48 | 1.06 | 0.33 | 1.57E-02 | |
| UN027266 | GRAS family transcription factor | 3.88 | 2.50 | 3.19 | 26.61 | 28.17 | 27.39 | 8.59 | 2.08E-22 | |
| UN027268 | GRAS family transcription factor isoform 1 | 2.75 | 0.94 | 1.84 | 11.93 | 10.71 | 11.32 | 6.14 | 2.35E-05 | |
| UN002238 | GRAS family transcription factor | 4.74 | 4.90 | 4.82 | 21.47 | 23.33 | 22.40 | 4.65 | 3.84E-14 | |
| UN005805 | GRAS family transcription factor | 2.75 | 2.07 | 2.41 | 5.98 | 5.69 | 5.83 | 2.42 | 6.62E-03 | |
| UN012382 | BEST Arabidopsis thaliana protein match is: transcription factor-related | 2.40 | 2.46 | 2.43 | 13.19 | 16.79 | 14.99 | 6.17 | 4.45E-15 | |
| UN034746 | Nuclear transcription factor Y subunit A-3, putative | 0.36 | 0.11 | 0.23 | 1.45 | 1.43 | 1.44 | 6.13 | 1.03E-03 | |
| UN034745 | Nuclear transcription factor Y subunit A-3, putative | 0.62 | 0.38 | 0.50 | 2.63 | 3.32 | 2.97 | 5.95 | 1.65E-06 | |
| UN034748 | Nuclear transcription factor Y subunit A-3, putative | 0.42 | 0.12 | 0.27 | 1.54 | 1.61 | 1.57 | 5.83 | 8.75E-04 | |
| UN034747 | Nuclear transcription factor Y subunit A-3, putative | 0.69 | 0.38 | 0.53 | 2.72 | 3.51 | 3.11 | 5.82 | 1.53E-06 | |
| UN052138 | Nuclear transcription factor Y subunit A-3 | 2.70 | 2.60 | 2.65 | 12.99 | 11.57 | 12.28 | 4.63 | 1.93E-13 | |
| UN002256 | Nuclear transcription factor Y subunit A-3, putative | 2.65 | 1.87 | 2.26 | 7.23 | 6.16 | 6.70 | 2.96 | 1.48E-03 | |
| UN052134 | Nuclear transcription factor Y subunit A-3, putative | 4.12 | 2.59 | 3.35 | 9.83 | 8.53 | 9.18 | 2.74 | 3.39E-05 | |
| UN052140 | Nuclear transcription factor Y subunit A-3, putative | 4.41 | 2.67 | 3.54 | 10.03 | 9.04 | 9.54 | 2.69 | 4.14E-05 | |
| UN052135 | Nuclear transcription factor Y subunit A-3, putative | 3.78 | 2.29 | 3.04 | 8.53 | 7.54 | 8.04 | 2.65 | 1.32E-04 | |
| UN052137 | Nuclear transcription factor Y subunit A-3, putative | 3.46 | 2.31 | 2.88 | 8.01 | 7.22 | 7.62 | 2.64 | 1.44E-04 | |
| UN052139 | Nuclear transcription factor Y subunit A-3, putative | 3.75 | 2.45 | 3.10 | 8.35 | 7.78 | 8.06 | 2.60 | 2.58E-05 | |
| UN052141 | Nuclear transcription factor Y subunit A-3, putative | 3.91 | 2.50 | 3.21 | 8.47 | 8.10 | 8.29 | 2.59 | 3.19E-05 | |
| UN052136 | Nuclear transcription factor Y subunit A-3, putative | 3.62 | 2.32 | 2.97 | 7.84 | 7.44 | 7.64 | 2.57 | 4.98E-05 | |
| UN048916 | Nuclear transcription factor Y subunit C8 | 3.36 | 3.55 | 3.46 | 7.41 | 7.55 | 7.48 | 2.16 | 1.46E-03 | |
| UN028776 | Nuclear transcription factor Y subunit B-10 | 18.13 | 19.57 | 18.85 | 40.02 | 39.18 | 39.60 | 2.10 | 4.88E-04 | |
| UN028777 | Nuclear transcription factor Y subunit B-10 | 15.09 | 15.38 | 15.23 | 31.90 | 30.41 | 31.16 | 2.04 | 1.58E-03 | |
| UN002065 | Nuclear transcription factor y subunit b-3 | 59.94 | 63.63 | 61.78 | 28.85 | 26.93 | 27.89 | 0.45 | 1.82E-13 | |
| UN039715 | PLATZ transcription factor family protein | 0.77 | 0.56 | 0.67 | 3.86 | 3.95 | 3.91 | 5.87 | 3.31E-09 | |
| UN039714 | PLATZ transcription factor family protein | 1.29 | 1.24 | 1.27 | 7.13 | 7.43 | 7.28 | 5.75 | 4.98E-14 | |
| UN039717 | PLATZ transcription factor family protein | 1.20 | 1.02 | 1.11 | 6.04 | 6.65 | 6.35 | 5.72 | 4.61E-13 | |
| UN039716 | PLATZ transcription factor | 0.66 | 0.50 | 0.58 | 2.37 | 2.99 | 2.68 | 4.62 | 8.51E-06 | |
| UN021990 | PLATZ transcription factor family protein | 16.61 | 12.42 | 14.52 | 5.82 | 4.41 | 5.12 | 0.35 | 1.32E-12 | |
| UN003556 | PLATZ transcription factor family protein | 14.48 | 13.35 | 13.91 | 2.85 | 5.09 | 3.97 | 0.29 | 2.91E-15 | |
| UN011936 | GATA transcription factor 9 | 4.59 | 4.73 | 4.66 | 21.69 | 20.63 | 21.16 | 4.54 | 1.76E-16 | |
| UN052300 | GATA transcription factor 15-like protein | 3.12 | 3.80 | 3.46 | 10.68 | 11.30 | 10.99 | 3.18 | 4.02E-05 | |
| UN086762 | GATA transcription factor 5 | 1.18 | 1.42 | 1.30 | 4.19 | 3.37 | 3.78 | 2.91 | 9.20E-04 | |
| UN053037 | GATA transcription factor 15-like protein | 1.96 | 2.98 | 2.47 | 5.56 | 5.55 | 5.55 | 2.25 | 9.08E-03 | |
| UN053036 | GATA transcription factor 15, putative | 2.44 | 2.77 | 2.60 | 5.89 | 5.54 | 5.71 | 2.19 | 3.14E-02 | |
| UN035080 | GATA transcription factor 21 | 29.68 | 34.81 | 32.25 | 13.31 | 15.23 | 14.27 | 0.44 | 4.60E-06 | |
| UN035079 | GATA transcription factor 21 | 17.46 | 22.76 | 20.11 | 7.54 | 7.73 | 7.63 | 0.38 | 4.54E-04 | |
| UN043089 | Winged-helix DNA-binding transcription factor family protein, putative | 1.74 | 1.93 | 1.83 | 5.41 | 6.08 | 5.75 | 3.13 | 3.19E-03 | |
| UN016743 | Winged-helix DNA-binding transcription factor family protein, putative | 2.03 | 1.71 | 1.87 | 4.12 | 5.30 | 4.71 | 2.52 | 4.29E-03 | |
| UN016744 | Winged-helix DNA-binding transcription factor family protein, putative | 2.43 | 1.88 | 2.16 | 4.74 | 6.00 | 5.37 | 2.49 | 4.38E-03 | |
| UN018713 | TCP family transcription factor family protein | 4.44 | 3.90 | 4.17 | 10.21 | 9.13 | 9.67 | 2.32 | 9.23E-05 | |
| UN089567 | TCP family transcription factor, putative | 4.78 | 4.74 | 4.76 | 10.82 | 11.16 | 10.99 | 2.31 | 5.62E-04 | |
| UN018714 | TCP family transcription factor family protein | 5.10 | 4.45 | 4.78 | 11.46 | 10.54 | 11.00 | 2.30 | 1.41E-04 | |
| UN010717 | TCP family transcription factor family protein | 2.51 | 2.77 | 2.64 | 1.02 | 1.22 | 1.12 | 0.42 | 3.44E-04 | |
| UN056184 | Transcription factor TCP4-like protein | 18.56 | 18.88 | 18.72 | 8.56 | 7.30 | 7.93 | 0.42 | 1.32E-12 | |
| UN056185 | Transcription factor TCP4-like protein | 7.31 | 8.72 | 8.02 | 3.34 | 3.39 | 3.37 | 0.42 | 7.37E-06 | |
| UN056181 | Transcription factor TCP4-like protein | 23.54 | 23.85 | 23.70 | 10.78 | 8.55 | 9.66 | 0.41 | 3.92E-14 | |
| UN056186 | Transcription factor TCP4-like protein | 19.04 | 18.86 | 18.95 | 8.20 | 7.17 | 7.68 | 0.41 | 2.37E-14 | |
| UN056187 | Transcription factor TCP4-like protein | 25.34 | 25.63 | 25.48 | 10.85 | 8.78 | 9.81 | 0.39 | 2.48E-16 | |
| UN056188 | Transcription factor TCP4-like protein | 14.20 | 15.54 | 14.87 | 6.41 | 5.07 | 5.74 | 0.39 | 5.50E-11 | |
| UN056182 | Transcription factor TCP4-like protein | 11.12 | 11.59 | 11.36 | 4.37 | 4.34 | 4.36 | 0.38 | 8.57E-13 | |
| UN056183 | Transcription factor TCP4-like protein | 19.11 | 20.14 | 19.62 | 7.73 | 6.36 | 7.04 | 0.36 | 1.95E-16 | |
| UN079459 | Transcription factor TCP4 | 11.22 | 12.33 | 11.78 | 3.92 | 3.52 | 3.72 | 0.32 | 1.41E-14 | |
| UN083406 | PH-response transcription factor pacC/RIM101 isoform 1 | 89.38 | 79.35 | 84.36 | 27.64 | 29.93 | 28.79 | 0.34 | 6.44E-21 | |
| UN018720 | General transcription factor 2-related zinc finger protein | 8.09 | 8.73 | 8.41 | 4.65 | 3.31 | 3.98 | 0.47 | 2.01E-07 | |
| UN036149 | sequence-specific DNA binding transcription factors | 0.33 | 0.21 | 0.27 | 25.57 | 22.30 | 23.94 | 88.65 | 1.85E-39 | |
| UN024560 | kow domain-containing transcription factor 1 | 12.58 | 8.54 | 10.56 | 26.35 | 26.15 | 26.25 | 2.49 | 6.33E-06 | |
