# Supplementary material for: NAA and 6-BA promote accumulation of oleanolic acid by JA regulation in Achyranthes bidentata Bl
Source: PLoS One. 2020 Feb 27;15(2):e0229490. doi: 10.1371/journal.pone.0229490 (PMC7046271; doi:10.1371/journal.pone.0229490)
Supplement: S4 Table — (DOCX) [file pone.0229490.s008.docx]

**S4 Table. List of candidate genes encoding enzymes in the oleanolic acid biosynthesis pathways.**

| **ID** | **Description** | **L_CK vs L_T** | | | | | | | |
| --- | --- | --- | --- | --- | --- | --- | --- | --- | --- |
|  |  | **L_CK_rep1** | **L_CK_rep2** | **mean** | **L_T_rep1** | **L_T_rep2** | **mean** | **ratio** | **adjust p** |
| UN001593 | Hydroxymethylglutaryl-CoA synthase-like protein | 11.76 | 11.03 | 11.39 | 66.74 | 46.43 | 56.58 | 4.97 | 1.59E-04 |
| UN083791 | Hydroxymethylglutaryl-CoA synthase-like protein | 4.25 | 5.49 | 4.87 | 37.56 | 21.30 | 29.43 | 6.04 | 1.10E-02 |
| UN087391 | Hydroxymethylglutaryl-CoA synthase-like protein | 2.95 | 3.63 | 3.29 | 6.72 | 5.42 | 6.07 | 1.84 | 4.88E-01 |
| UN010524 | 3-hydroxy-3-methylglutaryl coenzyme A reductase | 2.44 | 2.19 | 2.31 | 0.86 | 0.74 | 0.80 | 0.35 | 8.60E-08 |
| UN018526 | 3-hydroxy-3-methylglutaryl coenzyme A reductase | 0.97 | 0.80 | 0.89 | 1.25 | 1.33 | 1.29 | 1.46 | 9.32E-01 |
| UN045093 | 3-hydroxy-3-methylglutaryl coenzyme A reductase | 6.44 | 5.01 | 5.72 | 218.81 | 158.08 | 188.44 | 32.92 | 1.11E-14 |
| UN045094 | 3-hydroxy-3-methylglutaryl coenzyme A reductase | 6.49 | 5.06 | 5.78 | 215.29 | 155.37 | 185.33 | 32.09 | 1.85E-14 |
| UN045095 | 3-hydroxy-3-methylglutaryl coenzyme A reductase | 18.34 | 15.79 | 17.06 | 342.18 | 268.19 | 305.19 | 17.88 | 8.48E-19 |
| UN045096 | 3-hydroxy-3-methylglutaryl coenzyme A reductase | 0.18 | 0.23 | 0.21 | 125.19 | 80.49 | 102.84 | 501.66 | 1.07E-15 |
| UN057554 | 3-hydroxy-3-methylglutaryl coenzyme A reductase | 5.91 | 4.86 | 5.38 | 19.40 | 16.38 | 17.89 | 3.32 | 1.34E-09 |
| UN057555 | 3-hydroxy-3-methylglutaryl coenzyme A reductase | 3.90 | 3.27 | 3.58 | 12.49 | 10.62 | 11.55 | 3.22 | 6.44E-10 |
| UN083480 | 3-hydroxy-3-methylglutaryl coenzyme A reductase | 0.12 | 0.46 | 0.29 | 74.49 | 46.53 | 60.51 | 208.66 | 1.41E-11 |
| UN090588 | 3-hydroxy-3-methylglutaryl-coenzyme A reductase | 0.30 | 0.45 | 0.38 | 0.43 | 0.65 | 0.54 | 1.44 | 1.00E+00 |
| UN018870 | mevalonate kinase | 3.00 | 2.59 | 2.79 | 5.54 | 3.76 | 4.65 | 1.66 | 4.88E-01 |
| UN022998 | Mevalonate kinase family protein | 1.22 | 0.75 | 0.98 | 1.35 | 1.25 | 1.30 | 1.32 | 1.00E+00 |
| UN046667 | Phosphomevalonate kinase | 9.12 | 8.39 | 8.75 | 32.65 | 25.66 | 29.16 | 3.33 | 8.48E-06 |
| UN046669 | Phosphomevalonate kinase | 7.67 | 6.09 | 6.88 | 16.51 | 15.47 | 15.99 | 2.32 | 8.10E-05 |
| UN046670 | Phosphomevalonate kinase | 6.80 | 6.27 | 6.54 | 41.39 | 31.91 | 36.65 | 5.61 | 1.91E-08 |
| UN092680 | Diphosphomevalonate decarboxylase, putative | 11.54 | 10.91 | 11.22 | 223.28 | 146.57 | 184.93 | 16.47 | 3.72E-07 |
| UN099102 | Isopentenyl diphosphate isomerase | 12.49 | 12.31 | 12.40 | 10.83 | 8.69 | 9.76 | 0.79 | 9.64E-02 |
| UN082296 | Isopentenyl-diphosphate delta-isomerase | 16.77 | 13.92 | 15.34 | 19.04 | 18.15 | 18.59 | 1.21 | 1.00E+00 |
| UN012145 | Isopentenyl-diphosphate Delta-isomerase II | 59.88 | 61.22 | 60.55 | 644.49 | 498.52 | 571.50 | 9.44 | 2.17E-12 |
| UN046046 | Geranyl diphosphate synthase | 8.49 | 7.93 | 8.21 | 5.30 | 7.07 | 6.19 | 0.75 | 1.03E-02 |
| UN046047 | Geranyl diphosphate synthase | 10.22 | 8.91 | 9.57 | 6.93 | 8.94 | 7.93 | 0.83 | 3.67E-02 |
| UN046049 | Geranyl diphosphate synthase | 6.26 | 5.20 | 5.73 | 4.78 | 6.52 | 5.65 | 0.99 | 6.79E-01 |
| UN078924 | Geranylgeranyl diphosphate synthase 1 | 42.69 | 46.68 | 44.69 | 20.87 | 22.54 | 21.70 | 0.49 | 1.26E-09 |
| UN091844 | Geranylgeranyl pyrophosphate synthase | 0.15 | 0.38 | 0.27 | 0.33 | 0.32 | 0.33 | 1.23 | 1.00E+00 |
| UN004205 | Farnesyl pyrophosphate synthase | 0.13 | 0.23 | 0.18 | 2.40 | 0.99 | 1.69 | 9.42 | 8.05E-02 |
| UN026373 | Farnesyl diphosphate synthase | 0.25 | 0.44 | 0.34 | 3.48 | 1.36 | 2.42 | 7.01 | 1.58E-01 |
| UN026375 | Farnesyl diphosphate synthase | 0.15 | 0.20 | 0.17 | 1.37 | 0.83 | 1.10 | 6.29 | 1.20E-01 |
| UN026374 | Farnesyl pyrophosphate synthase | 0.15 | 0.37 | 0.26 | 2.80 | 1.04 | 1.92 | 7.38 | 1.63E-01 |
| UN026377 | Farnesyl pyrophosphate synthase | 0.09 | 0.24 | 0.16 | 1.58 | 0.69 | 1.14 | 6.88 | 1.11E-01 |
| UN026376 | Farnesyl pyrophosphate synthase 1 | 0.15 | 0.19 | 0.17 | 1.50 | 0.72 | 1.11 | 6.53 | 1.02E-01 |
| UN092583 | Farnesyl pyrophosphate synthase 1 | 12.28 | 12.32 | 12.30 | 21.19 | 21.05 | 21.12 | 1.72 | 4.20E-02 |
| UN041060 | Farnesyl-diphosphate synthase | 9.03 | 11.86 | 10.45 | 342.73 | 276.89 | 309.81 | 29.66 | 1.61E-29 |
| UN045623 | Squalene monooxygenase | 3.07 | 3.87 | 3.47 | 97.15 | 67.43 | 82.29 | 23.71 | 1.15E-10 |
| UN045637 | Squalene monooxygenase, putative | 0.13 | 0.17 | 0.15 | 3.57 | 3.17 | 3.37 | 22.47 | 3.81E-04 |
| UN045622 | Squalene monooxygenase, putative, expressed | 1.98 | 2.41 | 2.20 | 62.49 | 44.07 | 53.28 | 24.27 | 1.56E-11 |
| UN011483 | Squalene synthase | 0.00 | 0.00 | 0.00 | 2.85 | 3.37 | 3.11 | 311.00 | 1.49E-20 |
| UN043163 | Squalene synthase | 17.03 | 17.92 | 17.48 | 190.72 | 138.34 | 164.53 | 9.42 | 1.67E-08 |
| UN043164 | Squalene synthase | 16.24 | 17.48 | 16.86 | 183.52 | 133.34 | 158.43 | 9.40 | 1.46E-08 |
| Contig39705 | Beta-amyrin synthase | 0.79 | 0.65 | 0.72 | 9.13 | 6.72 | 7.92 | 10.96 | 4.60E-01 |
| Contig935 | Beta-amyrin synthase | 3.20 | 2.66 | 2.93 | 274.27 | 202.73 | 238.50 | 81.32 | 5.72E-65 |
| Contig50904 | Beta-amyrin synthase | 0.20 | 0.41 | 0.31 | 0.03 | 0.02 | 0.02 | 0.08 | 1.00E+00 |
| Contig7815 | Cycloartenol synthase protein | 13.17 | 6.71 | 9.94 | 4.26 | 5.24 | 4.75 | 0.48 | 1.00E+00 |
| First_Contig45 | Cycloartenol synthase 1 isoform 1 | 20.70 | 11.86 | 16.28 | 17.17 | 14.16 | 15.67 | 0.96 | 1.00E+00 |
| UN001948 | Glycosyltransferase | 4.53 | 4.24 | 4.38 | 10.33 | 10.17 | 10.25 | 2.34 | 6.27E-03 |
| UN024570 | Glycosyltransferase family 2 protein | 0.41 | 0.43 | 0.42 | 0.68 | 0.88 | 0.78 | 1.86 | 6.01E-01 |
| UN083908 | Glycosyltransferase family 4 protein, putative alpha-1,3-mannosyltransferase ALG2 | 29.00 | 28.58 | 28.79 | 22.62 | 21.48 | 22.05 | 0.77 | 2.49E-03 |
| UN012643 | Glycosyltransferase family 61 protein | 17.38 | 16.58 | 16.98 | 40.03 | 46.29 | 43.16 | 2.54 | 2.68E-07 |
| UN023100 | Glycosyltransferase family 61 protein | 0.34 | 0.31 | 0.33 | 2.14 | 2.72 | 2.43 | 7.48 | 5.57E-06 |
| UN046537 | Glycosyltransferase family 61 protein | 7.35 | 6.82 | 7.08 | 24.97 | 21.57 | 23.27 | 3.28 | 1.74E-10 |
| UN046538 | Glycosyltransferase family 61 protein | 1.51 | 1.03 | 1.27 | 11.45 | 11.81 | 11.63 | 9.16 | 4.36E-26 |
| UN046539 | Glycosyltransferase family 61 protein | 12.16 | 11.31 | 11.73 | 35.74 | 32.39 | 34.06 | 2.90 | 5.29E-09 |
| UN046540 | Glycosyltransferase family 61 protein | 4.09 | 3.43 | 3.76 | 15.92 | 16.54 | 16.23 | 4.32 | 2.01E-14 |
| UN090951 | Glycosyltransferase family 61 protein | 1.06 | 1.28 | 1.17 | 0.73 | 0.92 | 0.82 | 0.71 | 3.22E-01 |
| UN099204 | Glycosyltransferase family 61 protein | 0.00 | 0.00 | 0.00 | 1.74 | 1.34 | 1.54 | 154.00 | 9.94E-12 |
| UN011751 | Glycosyltransferase family 61 protein, putative | 2.51 | 1.97 | 2.24 | 4.94 | 5.23 | 5.08 | 2.27 | 5.34E-03 |
| UN092602 | Glycosyltransferase family 61 protein, putative | 0.12 | 0.23 | 0.17 | 2.22 | 2.64 | 2.43 | 13.89 | 1.07E-09 |
| UN007983 | Glycosyltransferase, putative | 3.01 | 2.97 | 2.99 | 13.56 | 14.98 | 14.27 | 4.77 | 7.27E-18 |
| UN012644 | Glycosyltransferase, putative | 23.26 | 21.12 | 22.19 | 58.89 | 67.28 | 63.09 | 2.84 | 1.50E-09 |
| UN017938 | Glycosyltransferase, putative | 0.38 | 0.20 | 0.29 | 1.19 | 1.47 | 1.33 | 4.59 | 1.55E-01 |
| UN055179 | Glycosyltransferase, putative | 2.56 | 3.24 | 2.90 | 7.11 | 7.72 | 7.42 | 2.56 | 1.79E-05 |
| UN088789 | Glycosyltransferase, putative | 0.89 | 1.80 | 1.34 | 3.11 | 3.54 | 3.33 | 2.47 | 2.92E-01 |
| UN005444 | UDP glycosyltransferase | 30.08 | 33.91 | 31.99 | 8.09 | 10.80 | 9.45 | 0.30 | 8.20E-12 |
| UN021810 | UDP glycosyltransferase | 1.05 | 1.04 | 1.04 | 0.75 | 0.84 | 0.79 | 0.76 | 3.58E-01 |
| UN028158 | UDP glycosyltransferase | 1.24 | 1.87 | 1.56 | 0.12 | 0.33 | 0.23 | 0.14 | 1.59E-02 |
| UN002025 | UDP-glycosyltransferase | 16.78 | 14.40 | 15.59 | 9.44 | 12.49 | 10.96 | 0.70 | 5.75E-04 |
| UN003915 | UDP-glycosyltransferase | 25.14 | 24.88 | 25.01 | 4.36 | 7.03 | 5.70 | 0.23 | 8.98E-28 |
| UN004420 | UDP-glycosyltransferase | 2.03 | 2.11 | 2.07 | 1.69 | 1.72 | 1.71 | 0.82 | 2.44E-01 |
| UN004529 | UDP-glycosyltransferase | 42.12 | 47.95 | 45.03 | 17.29 | 18.53 | 17.91 | 0.40 | 9.85E-09 |
| UN005368 | UDP-glycosyltransferase | 2.83 | 3.08 | 2.96 | 1.15 | 1.27 | 1.21 | 0.41 | 1.91E-05 |
| UN009425 | UDP-glycosyltransferase | 15.19 | 14.97 | 15.08 | 15.82 | 13.06 | 14.44 | 0.96 | 2.13E-01 |
| UN009878 | UDP-glycosyltransferase | 23.33 | 17.56 | 20.45 | 23.04 | 23.18 | 23.11 | 1.13 | 7.46E-01 |
| UN012908 | UDP-glycosyltransferase | 0.10 | 0.00 | 0.05 | 0.67 | 0.76 | 0.72 | 14.30 | 2.64E-01 |
| UN014308 | UDP-glycosyltransferase | 0.84 | 0.25 | 0.54 | 0.14 | 0.63 | 0.39 | 0.71 | 7.95E-01 |
| UN016642 | UDP-glycosyltransferase | 0.22 | 0.22 | 0.22 | 1.18 | 1.44 | 1.31 | 5.95 | 9.78E-04 |
| UN017109 | UDP-glycosyltransferase | 0.74 | 1.04 | 0.89 | 0.66 | 0.67 | 0.67 | 0.75 | 4.66E-01 |
| UN017110 | UDP-glycosyltransferase | 0.76 | 0.72 | 0.74 | 0.78 | 0.60 | 0.69 | 0.93 | 6.84E-01 |
| UN017514 | UDP-glycosyltransferase | 0.77 | 0.79 | 0.78 | 0.00 | 0.25 | 0.12 | 0.16 | 7.54E-02 |
| UN018268 | UDP-glycosyltransferase | 2.38 | 3.78 | 3.08 | 1.40 | 1.67 | 1.53 | 0.50 | 1.18E-01 |
| UN019911 | UDP-glycosyltransferase | 1.28 | 1.10 | 1.19 | 0.32 | 0.46 | 0.39 | 0.33 | 6.17E-03 |
| UN023002 | UDP-glycosyltransferase | 0.19 | 0.21 | 0.20 | 6.52 | 5.01 | 5.76 | 28.82 | 1.56E-16 |
| UN025158 | UDP-glycosyltransferase | 77.93 | 92.39 | 85.16 | 20.97 | 25.82 | 23.39 | 0.27 | 4.89E-09 |
| UN028157 | UDP-glycosyltransferase | 12.54 | 13.73 | 13.13 | 2.96 | 3.36 | 3.16 | 0.24 | 6.56E-19 |
| UN028160 | UDP-glycosyltransferase | 7.07 | 7.52 | 7.29 | 1.67 | 2.41 | 2.04 | 0.28 | 1.08E-06 |
| UN033160 | UDP-glycosyltransferase | 7.24 | 8.52 | 7.88 | 2.60 | 2.23 | 2.42 | 0.31 | 2.18E-08 |
| UN033413 | UDP-glycosyltransferase | 17.59 | 28.01 | 22.80 | 1.62 | 1.64 | 1.63 | 0.07 | 2.90E-05 |
| UN033843 | UDP-glycosyltransferase | 0.00 | 0.65 | 0.33 | 0.00 | 0.00 | 0.00 | 0.03 | 7.58E-01 |
| UN037205 | UDP-glycosyltransferase | 13.89 | 8.37 | 11.13 | 5.02 | 3.13 | 4.07 | 0.37 | 1.55E-03 |
| UN041012 | UDP-glycosyltransferase | 7.74 | 7.04 | 7.39 | 0.81 | 0.48 | 0.65 | 0.09 | 3.58E-19 |
| UN043472 | UDP-glycosyltransferase | 0.24 | 0.20 | 0.22 | 69.45 | 42.34 | 55.90 | 254.07 | 2.13E-12 |
| UN048394 | UDP-glycosyltransferase | 3.14 | 1.92 | 2.53 | 6.74 | 7.79 | 7.27 | 2.87 | 1.59E-04 |
| UN048395 | UDP-glycosyltransferase | 3.08 | 2.06 | 2.57 | 6.30 | 6.64 | 6.47 | 2.52 | 2.22E-04 |
| UN059970 | UDP-glycosyltransferase | 20.88 | 24.85 | 22.87 | 4.57 | 5.67 | 5.12 | 0.22 | 2.81E-10 |
| UN059971 | UDP-glycosyltransferase | 21.09 | 25.34 | 23.21 | 4.46 | 5.69 | 5.07 | 0.22 | 8.51E-10 |
| UN068628 | UDP-glycosyltransferase | 4.70 | 3.88 | 4.29 | 0.84 | 1.25 | 1.04 | 0.24 | 2.57E-09 |
| UN075609 | UDP-glycosyltransferase | 46.16 | 53.81 | 49.98 | 36.54 | 42.49 | 39.52 | 0.79 | 3.80E-02 |
| UN076022 | UDP-glycosyltransferase | 2.46 | 2.08 | 2.27 | 5.20 | 4.64 | 4.92 | 2.17 | 2.66E-02 |
| UN080613 | UDP-glycosyltransferase | 39.79 | 38.90 | 39.34 | 9.81 | 11.71 | 10.76 | 0.27 | 3.07E-25 |
| UN083551 | UDP-glycosyltransferase | 6.74 | 4.80 | 5.77 | 0.77 | 0.46 | 0.61 | 0.11 | 2.54E-10 |
| UN086105 | UDP-glycosyltransferase | 2.07 | 2.00 | 2.04 | 26.51 | 28.76 | 27.64 | 13.58 | 1.01E-45 |
| UN086707 | UDP-glycosyltransferase | 0.00 | 0.00 | 0.00 | 1.10 | 1.41 | 1.25 | 125.50 | 4.78E-04 |
| UN089491 | UDP-glycosyltransferase | 8.86 | 11.31 | 10.09 | 1.52 | 1.55 | 1.54 | 0.15 | 1.18E-08 |
| UN093393 | UDP-glycosyltransferase | 1.87 | 1.96 | 1.92 | 1.10 | 1.09 | 1.10 | 0.57 | 2.69E-02 |
| UN100506 | UDP-glycosyltransferase | 0.00 | 0.00 | 0.00 | 8.49 | 9.25 | 8.87 | 887.00 | 3.24E-53 |
| UN001798 | UDP-glycosyltransferase 1 | 0.67 | 0.68 | 0.68 | 0.53 | 0.77 | 0.65 | 0.96 | 9.29E-01 |
| UN002826 | UDP-glycosyltransferase 1 | 0.30 | 0.30 | 0.30 | 4.38 | 6.06 | 5.22 | 17.40 | 8.80E-14 |
| UN003228 | UDP-glycosyltransferase 1 | 3.86 | 3.67 | 3.76 | 5.57 | 4.62 | 5.10 | 1.35 | 8.89E-01 |
| UN005853 | UDP-glycosyltransferase 1 | 1.89 | 1.63 | 1.76 | 3.43 | 2.53 | 2.98 | 1.69 | 3.75E-01 |
| UN006527 | UDP-glycosyltransferase 1 | 10.78 | 20.44 | 15.61 | 10.85 | 4.55 | 7.70 | 0.49 | 3.38E-01 |
| UN014129 | UDP-glycosyltransferase 1 | 0.06 | 0.08 | 0.07 | 0.63 | 0.74 | 0.69 | 9.79 | 1.27E-01 |
| UN014130 | UDP-glycosyltransferase 1 | 0.42 | 0.24 | 0.33 | 2.12 | 3.23 | 2.67 | 8.11 | 1.98E-05 |
| UN015104 | UDP-glycosyltransferase 1 | 3.69 | 2.62 | 3.16 | 0.69 | 0.95 | 0.82 | 0.26 | 6.37E-08 |
| UN016808 | UDP-glycosyltransferase 1 | 2.02 | 1.87 | 1.95 | 0.66 | 0.52 | 0.59 | 0.30 | 2.91E-05 |
| UN025881 | UDP-glycosyltransferase 1 | 15.19 | 16.42 | 15.80 | 10.15 | 10.60 | 10.38 | 0.66 | 1.09E-04 |
| UN030472 | UDP-glycosyltransferase 1 | 2.42 | 2.20 | 2.31 | 0.15 | 0.05 | 0.10 | 0.04 | 6.34E-11 |
| UN033161 | UDP-glycosyltransferase 1 | 19.42 | 20.61 | 20.02 | 5.80 | 6.62 | 6.21 | 0.31 | 7.81E-19 |
| UN033162 | UDP-glycosyltransferase 1 | 5.68 | 6.08 | 5.88 | 1.35 | 1.24 | 1.29 | 0.22 | 1.88E-15 |
| UN033411 | UDP-glycosyltransferase 1 | 2.46 | 2.43 | 2.45 | 9.30 | 7.59 | 8.45 | 3.45 | 5.11E-07 |
| UN033412 | UDP-glycosyltransferase 1 | 1.45 | 1.32 | 1.39 | 6.26 | 4.89 | 5.57 | 4.03 | 5.34E-05 |
| UN038307 | UDP-glycosyltransferase 1 | 15.49 | 18.70 | 17.09 | 2.29 | 5.20 | 3.75 | 0.22 | 5.08E-08 |
| UN038308 | UDP-glycosyltransferase 1 | 20.33 | 23.49 | 21.91 | 3.06 | 6.12 | 4.59 | 0.21 | 2.27E-11 |
| UN038422 | UDP-glycosyltransferase 1 | 1.27 | 1.27 | 1.27 | 3.37 | 2.00 | 2.69 | 2.11 | 3.22E-01 |
| UN038423 | UDP-glycosyltransferase 1 | 2.13 | 1.50 | 1.81 | 1.01 | 0.69 | 0.85 | 0.47 | 6.68E-02 |
| UN038424 | UDP-glycosyltransferase 1 | 1.57 | 1.28 | 1.42 | 1.06 | 0.66 | 0.86 | 0.60 | 1.44E-01 |
| UN038425 | UDP-glycosyltransferase 1 | 3.26 | 4.21 | 3.73 | 2.26 | 1.97 | 2.11 | 0.57 | 1.46E-02 |
| UN052240 | UDP-glycosyltransferase 1 | 0.48 | 0.26 | 0.37 | 2.14 | 1.33 | 1.74 | 4.69 | 1.73E-02 |
| UN068623 | UDP-glycosyltransferase 1 | 1.57 | 1.47 | 1.52 | 0.97 | 0.70 | 0.83 | 0.55 | 1.45E-01 |
| UN068624 | UDP-glycosyltransferase 1 | 1.11 | 1.44 | 1.27 | 0.83 | 1.05 | 0.94 | 0.74 | 4.63E-01 |
| UN068625 | UDP-glycosyltransferase 1 | 1.39 | 1.74 | 1.56 | 1.32 | 1.09 | 1.21 | 0.77 | 4.12E-01 |
| UN079145 | UDP-glycosyltransferase 1 | 38.21 | 40.11 | 39.16 | 15.47 | 13.71 | 14.59 | 0.37 | 2.12E-17 |
| UN079297 | UDP-glycosyltransferase 1 | 0.00 | 0.00 | 0.00 | 3.51 | 3.24 | 3.38 | 337.50 | 1.67E-24 |
| UN081165 | UDP-glycosyltransferase 1 | 1.63 | 1.64 | 1.64 | 1.11 | 1.26 | 1.19 | 0.72 | 5.69E-01 |
| UN082365 | UDP-glycosyltransferase 1 | 9.28 | 7.21 | 8.24 | 7.68 | 7.81 | 7.75 | 0.94 | 1.92E-01 |
| UN082600 | UDP-glycosyltransferase 1 | 0.48 | 0.82 | 0.65 | 0.35 | 0.00 | 0.17 | 0.27 | 5.93E-01 |
| UN082614 | UDP-glycosyltransferase 1 | 2.06 | 0.93 | 1.50 | 0.68 | 1.28 | 0.98 | 0.66 | 4.08E-01 |
| UN085282 | UDP-glycosyltransferase 1 | 0.75 | 0.19 | 0.47 | 0.00 | 0.16 | 0.08 | 0.17 | 5.57E-01 |
| UN088526 | UDP-glycosyltransferase 1 | 0.18 | 0.00 | 0.09 | 0.00 | 0.19 | 0.10 | 1.06 | 1.00E+00 |
| UN092670 | UDP-glycosyltransferase 1 | 0.02 | 0.03 | 0.03 | 30.72 | 27.57 | 29.14 | 1165.80 | 8.52E-118 |
| UN094767 | UDP-glycosyltransferase 1 | 1.16 | 0.99 | 1.07 | 0.86 | 0.83 | 0.84 | 0.79 | 8.48E-01 |
| UN098072 | UDP-glycosyltransferase 1 | 4.01 | 5.12 | 4.56 | 1.32 | 1.79 | 1.56 | 0.34 | 1.02E-04 |
| UN000378 | UDP-glycosyltransferase 74 F1, putative | 5.11 | 6.79 | 5.95 | 1.08 | 1.19 | 1.14 | 0.19 | 3.53E-06 |
| UN016337 | UDP-glycosyltransferase 74 F1, putative | 1.47 | 1.79 | 1.63 | 0.95 | 1.09 | 1.02 | 0.63 | 7.79E-02 |
| UN068626 | UDP-glycosyltransferase 74 F1, putative | 4.13 | 4.61 | 4.37 | 1.09 | 1.85 | 1.47 | 0.34 | 1.05E-06 |
| UN068627 | UDP-glycosyltransferase 74 F1, putative | 5.69 | 7.42 | 6.55 | 1.66 | 1.53 | 1.60 | 0.24 | 1.00E-05 |
| UN068629 | UDP-glycosyltransferase 74 F1, putative | 5.11 | 4.84 | 4.97 | 0.65 | 1.77 | 1.21 | 0.24 | 1.82E-08 |
| UN089091 | UDP-glycosyltransferase 74 F1, putative | 91.60 | 85.41 | 88.50 | 26.42 | 30.23 | 28.33 | 0.32 | 1.34E-23 |
| UN068621 | UDP-glycosyltransferase 74E2 | 0.87 | 0.93 | 0.90 | 1.87 | 1.74 | 1.81 | 2.01 | 2.73E-01 |
| UN033410 | UDP-Glycosyltransferase superfamily protein | 2.99 | 2.76 | 2.88 | 7.80 | 6.65 | 7.22 | 2.51 | 8.84E-03 |
| UN033842 | UDP-Glycosyltransferase superfamily protein | 0.00 | 0.15 | 0.07 | 0.00 | 0.00 | 0.00 | 0.13 | 1.00E+00 |
| UN033844 | UDP-Glycosyltransferase superfamily protein | 0.14 | 0.00 | 0.07 | 0.76 | 1.02 | 0.89 | 12.71 | 3.16E-01 |
| UN044437 | UDP-Glycosyltransferase superfamily protein | 44.03 | 36.30 | 40.16 | 25.79 | 24.70 | 25.24 | 0.63 | 1.64E-06 |
| UN052239 | UDP-Glycosyltransferase superfamily protein | 3.39 | 3.38 | 3.38 | 4.22 | 3.68 | 3.95 | 1.17 | 9.68E-01 |
| UN078976 | UDP-Glycosyltransferase superfamily protein | 0.07 | 0.16 | 0.12 | 20.07 | 21.52 | 20.80 | 180.83 | 1.36E-85 |
| UN078978 | UDP-Glycosyltransferase superfamily protein | 16.30 | 14.31 | 15.30 | 20.17 | 19.42 | 19.80 | 1.29 | 9.70E-01 |
| UN080852 | UDP-Glycosyltransferase superfamily protein | 1.11 | 0.00 | 0.56 | 0.00 | 0.45 | 0.23 | 0.41 | 8.57E-01 |
| UN088360 | UDP-Glycosyltransferase superfamily protein | 0.00 | 0.69 | 0.34 | 0.20 | 0.00 | 0.10 | 0.29 | 9.46E-01 |
| UN090091 | UDP-Glycosyltransferase superfamily protein | 0.00 | 0.00 | 0.00 | 2.20 | 2.15 | 2.17 | 217.50 | 4.49E-14 |
| UN093143 | UDP-Glycosyltransferase superfamily protein | 9.45 | 12.17 | 10.81 | 0.84 | 1.71 | 1.27 | 0.12 | 3.80E-09 |
| UN098598 | UDP-Glycosyltransferase superfamily protein | 8.76 | 6.73 | 7.75 | 2.37 | 2.48 | 2.42 | 0.31 | 7.45E-14 |
| UN099232 | UDP-Glycosyltransferase superfamily protein | 8.56 | 6.45 | 7.51 | 3.81 | 3.85 | 3.83 | 0.51 | 1.14E-06 |
| UN099233 | UDP-Glycosyltransferase superfamily protein | 7.60 | 5.30 | 6.45 | 3.44 | 3.46 | 3.45 | 0.53 | 5.52E-05 |
| UN099599 | UDP-Glycosyltransferase superfamily protein | 0.40 | 0.51 | 0.46 | 0.44 | 0.49 | 0.46 | 1.02 | 1.00E+00 |
| UN001067 | UDP-Glycosyltransferase superfamily protein, putative | 5.97 | 5.02 | 5.49 | 4.63 | 4.97 | 4.80 | 0.87 | 1.27E-01 |
| UN009284 | UDP-Glycosyltransferase superfamily protein, putative | 4.32 | 3.38 | 3.85 | 10.28 | 13.63 | 11.96 | 3.11 | 1.77E-05 |
| UN009285 | UDP-Glycosyltransferase superfamily protein, putative | 10.45 | 7.69 | 9.07 | 26.01 | 35.11 | 30.56 | 3.37 | 2.91E-05 |
| UN013066 | UDP-Glycosyltransferase superfamily protein, putative | 2.90 | 3.19 | 3.04 | 2.34 | 2.61 | 2.47 | 0.81 | 2.43E-01 |
| UN013067 | UDP-Glycosyltransferase superfamily protein, putative | 2.36 | 2.68 | 2.52 | 2.14 | 2.17 | 2.16 | 0.86 | 2.95E-01 |
| UN014770 | UDP-Glycosyltransferase superfamily protein, putative | 1.85 | 1.68 | 1.77 | 0.36 | 0.35 | 0.35 | 0.20 | 2.28E-02 |
| UN014771 | UDP-Glycosyltransferase superfamily protein, putative | 1.10 | 0.95 | 1.02 | 0.20 | 0.20 | 0.20 | 0.20 | 7.60E-02 |
| UN026863 | UDP-Glycosyltransferase superfamily protein, putative | 0.73 | 0.68 | 0.71 | 0.78 | 0.79 | 0.79 | 1.11 | 1.00E+00 |
| UN030469 | UDP-Glycosyltransferase superfamily protein, putative | 3.64 | 2.59 | 3.12 | 1.67 | 0.98 | 1.32 | 0.43 | 3.26E-05 |
| UN030470 | UDP-Glycosyltransferase superfamily protein, putative | 3.14 | 2.24 | 2.69 | 1.93 | 1.20 | 1.56 | 0.58 | 1.44E-02 |
| UN038387 | UDP-Glycosyltransferase superfamily protein, putative | 1.06 | 0.50 | 0.78 | 0.75 | 0.72 | 0.73 | 0.94 | 9.40E-01 |
| UN038388 | UDP-Glycosyltransferase superfamily protein, putative | 2.18 | 3.52 | 2.85 | 5.22 | 4.92 | 5.07 | 1.78 | 2.64E-01 |
| UN038389 | UDP-Glycosyltransferase superfamily protein, putative | 5.63 | 4.75 | 5.19 | 1.82 | 2.01 | 1.91 | 0.37 | 1.35E-06 |
| UN038390 | UDP-Glycosyltransferase superfamily protein, putative | 2.26 | 3.63 | 2.94 | 5.49 | 5.09 | 5.29 | 1.80 | 2.49E-01 |
| UN038391 | UDP-Glycosyltransferase superfamily protein, putative | 2.85 | 4.47 | 3.66 | 6.55 | 6.10 | 6.32 | 1.73 | 3.00E-01 |
| UN038392 | UDP-Glycosyltransferase superfamily protein, putative | 2.73 | 3.76 | 3.25 | 6.39 | 5.76 | 6.07 | 1.87 | 6.71E-02 |
| UN038393 | UDP-Glycosyltransferase superfamily protein, putative | 2.43 | 3.40 | 2.92 | 5.82 | 5.10 | 5.46 | 1.87 | 9.07E-02 |
| UN046513 | UDP-Glycosyltransferase superfamily protein, putative | 186.60 | 181.46 | 184.03 | 41.28 | 42.91 | 42.09 | 0.23 | 1.66E-36 |
| UN062444 | UDP-Glycosyltransferase superfamily protein, putative | 12.95 | 8.78 | 10.86 | 29.96 | 28.18 | 29.07 | 2.68 | 5.01E-07 |
| UN083380 | UDP-Glycosyltransferase superfamily protein, putative | 11.74 | 9.71 | 10.73 | 3.98 | 4.60 | 4.29 | 0.40 | 2.92E-11 |
| UN097871 | UDP-Glycosyltransferase superfamily protein, putative | 2.53 | 1.87 | 2.20 | 0.95 | 0.84 | 0.90 | 0.41 | 1.40E-03 |
| UN097872 | UDP-Glycosyltransferase superfamily protein, putative | 3.47 | 3.02 | 3.25 | 1.31 | 0.77 | 1.04 | 0.32 | 1.47E-08 |
| UN001846 | Cytochrome P450 | 2.38 | 2.11 | 2.25 | 4.52 | 4.70 | 4.61 | 2.05 | 6.05E-03 |
| UN002460 | Cytochrome P450 | 9.18 | 7.28 | 8.23 | 3.02 | 4.88 | 3.95 | 0.48 | 8.46E-06 |
| UN003649 | Cytochrome P450 | 0.36 | 0.27 | 0.32 | 2.98 | 3.63 | 3.30 | 10.49 | 1.32E-13 |
| UN004198 | Cytochrome P450 | 5.80 | 4.05 | 4.92 | 2.11 | 2.15 | 2.13 | 0.43 | 1.04E-05 |
| UN004294 | Cytochrome P450 | 21.63 | 24.44 | 23.04 | 7.73 | 7.31 | 7.52 | 0.33 | 2.43E-11 |
| UN009560 | Cytochrome P450 | 101.59 | 87.72 | 94.66 | 53.91 | 58.40 | 56.16 | 0.59 | 4.92E-08 |
| UN016253 | Cytochrome P450 | 0.31 | 0.40 | 0.35 | 1.56 | 1.33 | 1.45 | 4.07 | 3.88E-03 |
| UN017759 | Cytochrome P450 | 0.27 | 0.00 | 0.14 | 0.00 | 0.00 | 0.00 | 0.07 | 9.26E-01 |
| UN017917 | Cytochrome P450 | 35.10 | 44.58 | 39.84 | 15.30 | 16.22 | 15.76 | 0.40 | 2.07E-04 |
| UN019160 | Cytochrome P450 | 0.49 | 0.18 | 0.33 | 2.89 | 4.62 | 3.75 | 11.21 | 2.86E-05 |
| UN020244 | Cytochrome P450 | 6.40 | 6.22 | 6.31 | 1.58 | 1.44 | 1.51 | 0.24 | 9.44E-12 |
| UN020245 | Cytochrome P450 | 5.62 | 5.16 | 5.39 | 1.17 | 1.12 | 1.15 | 0.21 | 1.43E-13 |
| UN022199 | Cytochrome P450 | 0.39 | 0.35 | 0.37 | 3.40 | 3.72 | 3.56 | 9.62 | 1.68E-10 |
| UN025591 | Cytochrome P450 | 4.24 | 2.49 | 3.37 | 0.82 | 2.19 | 1.50 | 0.45 | 4.21E-02 |
| UN032331 | Cytochrome P450 | 0.73 | 0.54 | 0.64 | 0.81 | 0.78 | 0.80 | 1.25 | 1.00E+00 |
| UN032445 | Cytochrome P450 | 1.63 | 1.70 | 1.67 | 3.54 | 3.74 | 3.64 | 2.19 | 2.50E-01 |
| UN033383 | Cytochrome P450 | 0.00 | 0.00 | 0.00 | 0.34 | 0.33 | 0.34 | 33.50 | 5.39E-01 |
| UN033386 | Cytochrome P450 | 0.00 | 0.00 | 0.00 | 0.16 | 0.00 | 0.08 | 8.00 | 1.00E+00 |
| UN033428 | Cytochrome P450 | 6.08 | 6.82 | 6.45 | 2.28 | 2.81 | 2.54 | 0.39 | 3.50E-09 |
| UN034087 | Cytochrome P450 | 6.43 | 8.44 | 7.43 | 5.05 | 7.01 | 6.03 | 0.81 | 3.31E-01 |
| UN036297 | Cytochrome P450 | 1.41 | 1.44 | 1.42 | 3.22 | 2.39 | 2.81 | 1.97 | 4.59E-01 |
| UN041123 | Cytochrome P450 | 0.08 | 0.00 | 0.04 | 0.67 | 0.32 | 0.49 | 12.38 | 3.12E-01 |
| UN041124 | Cytochrome P450 | 0.22 | 0.09 | 0.15 | 0.65 | 0.78 | 0.72 | 4.61 | 3.93E-01 |
| UN041563 | Cytochrome P450 | 5.06 | 2.60 | 3.83 | 7.13 | 8.47 | 7.80 | 2.04 | 1.36E-01 |
| UN041564 | Cytochrome P450 | 4.69 | 2.86 | 3.77 | 7.52 | 8.72 | 8.12 | 2.15 | 6.33E-03 |
| UN041566 | Cytochrome P450 | 3.10 | 1.54 | 2.32 | 4.61 | 5.12 | 4.87 | 2.10 | 9.58E-02 |
| UN041567 | Cytochrome P450 | 4.50 | 2.50 | 3.50 | 6.59 | 7.67 | 7.13 | 2.04 | 6.76E-02 |
| UN041568 | Cytochrome P450 | 3.07 | 1.34 | 2.21 | 4.05 | 4.50 | 4.28 | 1.94 | 3.56E-01 |
| UN041569 | Cytochrome P450 | 5.38 | 3.01 | 4.20 | 8.23 | 9.70 | 8.96 | 2.14 | 3.42E-02 |
| UN046250 | Cytochrome P450 | 0.22 | 0.25 | 0.23 | 0.84 | 1.32 | 1.08 | 4.60 | 1.18E-02 |
| UN046523 | Cytochrome P450 | 2.49 | 2.20 | 2.35 | 18.24 | 18.26 | 18.25 | 7.78 | 2.29E-28 |
| UN046524 | Cytochrome P450 | 2.46 | 1.60 | 2.03 | 15.60 | 15.43 | 15.52 | 7.64 | 3.72E-25 |
| UN052087 | Cytochrome P450 | 15.77 | 17.31 | 16.54 | 10.81 | 8.31 | 9.56 | 0.58 | 4.69E-05 |
| UN052088 | Cytochrome P450 | 4.73 | 4.71 | 4.72 | 4.57 | 4.23 | 4.40 | 0.93 | 3.12E-01 |
| UN073279 | Cytochrome P450 | 0.46 | 0.49 | 0.47 | 1.10 | 0.08 | 0.59 | 1.24 | 1.00E+00 |
| UN079342 | Cytochrome P450 | 0.00 | 0.00 | 0.00 | 0.96 | 0.67 | 0.81 | 81.50 | 2.13E-04 |
| UN082395 | Cytochrome P450 | 0.12 | 0.08 | 0.10 | 0.86 | 0.64 | 0.75 | 7.50 | 1.27E-01 |
| UN082587 | Cytochrome P450 | 7.57 | 6.28 | 6.93 | 898.40 | 733.10 | 815.75 | 117.80 | 8.61E-51 |
| UN084415 | Cytochrome P450 | 0.00 | 0.00 | 0.00 | 0.00 | 0.18 | 0.09 | 9.00 | 1.00E+00 |
| UN085763 | Cytochrome P450 | 0.14 | 0.11 | 0.12 | 0.77 | 0.88 | 0.82 | 6.60 | 7.54E-03 |
| UN086577 | Cytochrome P450 | 0.73 | 0.24 | 0.48 | 0.00 | 0.20 | 0.10 | 0.21 | 6.79E-01 |
| UN088796 | Cytochrome P450 | 0.14 | 0.00 | 0.07 | 0.16 | 0.15 | 0.15 | 2.21 | 1.00E+00 |
| UN089647 | Cytochrome P450 | 0.38 | 0.00 | 0.19 | 0.14 | 0.14 | 0.14 | 0.74 | 1.00E+00 |
| UN092575 | Cytochrome P450 | 20.50 | 18.17 | 19.34 | 4.22 | 4.35 | 4.29 | 0.22 | 1.05E-28 |
| UN096898 | Cytochrome P450 | 3.99 | 1.88 | 2.94 | 0.09 | 0.38 | 0.23 | 0.08 | 5.83E-04 |
| UN097966 | Cytochrome P450 | 0.30 | 0.26 | 0.28 | 0.68 | 0.33 | 0.51 | 1.80 | 1.00E+00 |
| UN099368 | Cytochrome P450 | 5.00 | 4.11 | 4.55 | 7.12 | 7.85 | 7.48 | 1.64 | 2.10E-01 |
| UN099369 | Cytochrome P450 | 4.09 | 3.27 | 3.68 | 5.38 | 5.78 | 5.58 | 1.52 | 4.58E-01 |
| UN099863 | Cytochrome P450 | 46.03 | 40.26 | 43.14 | 15.60 | 16.27 | 15.93 | 0.37 | 1.71E-18 |
| UN099864 | Cytochrome P450 | 41.81 | 36.89 | 39.35 | 14.37 | 14.92 | 14.64 | 0.37 | 4.21E-18 |
| UN100390 | Cytochrome P450 | 10.69 | 9.41 | 10.05 | 7.14 | 10.05 | 8.60 | 0.86 | 4.70E-02 |
| UN100539 | Cytochrome P450 | 1.13 | 0.98 | 1.05 | 0.69 | 0.78 | 0.73 | 0.70 | 3.36E-01 |
| UN032446 | Cytochrome P450 71B10 | 2.41 | 1.41 | 1.91 | 4.01 | 3.55 | 3.78 | 1.98 | 2.65E-01 |
| UN020908 | Cytochrome P450 family 709 protein | 0.00 | 0.00 | 0.00 | 0.16 | 0.15 | 0.15 | 15.50 | 1.00E+00 |
| UN020909 | Cytochrome P450 family 709 protein | 1.25 | 1.12 | 1.19 | 1.04 | 0.72 | 0.88 | 0.74 | 3.30E-01 |
| UN016767 | Cytochrome P450 family 71 protein | 2.27 | 2.59 | 2.43 | 1.28 | 3.33 | 2.31 | 0.95 | 8.49E-01 |
| UN017233 | Cytochrome P450 family 71 protein | 12.29 | 11.04 | 11.66 | 4.36 | 5.82 | 5.09 | 0.44 | 2.33E-05 |
| UN036441 | Cytochrome P450 family 71 protein | 8.01 | 6.00 | 7.00 | 36.00 | 38.21 | 37.11 | 5.30 | 1.77E-22 |
| UN082251 | Cytochrome P450 family 71 protein | 10.93 | 11.81 | 11.37 | 5.02 | 6.48 | 5.75 | 0.51 | 1.62E-07 |
| UN093792 | Cytochrome P450 family 71 protein | 0.00 | 0.00 | 0.00 | 9.02 | 9.53 | 9.27 | 927.50 | 8.83E-57 |
| UN029727 | Cytochrome P450 family 72 protein | 7.96 | 5.23 | 6.60 | 6.48 | 6.36 | 6.42 | 0.97 | 3.56E-01 |
| UN029728 | Cytochrome P450 family 72 protein | 7.51 | 4.76 | 6.13 | 6.08 | 5.97 | 6.03 | 0.98 | 4.46E-01 |
| UN029729 | Cytochrome P450 family 72 protein | 15.91 | 7.94 | 11.93 | 9.07 | 10.61 | 9.84 | 0.83 | 4.62E-01 |
| UN031510 | Cytochrome P450 family 72 protein | 6.50 | 6.94 | 6.72 | 0.63 | 1.97 | 1.30 | 0.19 | 1.54E-05 |
| UN033385 | Cytochrome P450 family 72 protein | 0.52 | 0.73 | 0.62 | 0.79 | 0.76 | 0.78 | 1.24 | 1.00E+00 |
| UN041125 | Cytochrome P450 family 72 protein | 0.00 | 0.06 | 0.03 | 1.09 | 1.58 | 1.33 | 44.50 | 2.08E-05 |
| UN041126 | Cytochrome P450 family 72 protein | 0.09 | 0.12 | 0.10 | 1.09 | 1.91 | 1.50 | 14.29 | 4.44E-04 |
| UN062031 | Cytochrome P450 family 72 protein | 289.24 | 283.67 | 286.46 | 76.67 | 69.57 | 73.12 | 0.26 | 8.26E-33 |
| UN062032 | Cytochrome P450 family 72 protein | 255.07 | 244.45 | 249.76 | 78.27 | 45.70 | 61.98 | 0.25 | 5.77E-34 |
| UN062033 | Cytochrome P450 family 72 protein | 195.65 | 186.88 | 191.26 | 58.94 | 35.87 | 47.41 | 0.25 | 6.76E-34 |
| UN062034 | Cytochrome P450 family 72 protein | 111.13 | 102.12 | 106.62 | 30.06 | 21.22 | 25.64 | 0.24 | 2.23E-34 |
| UN063351 | Cytochrome P450 family 72 protein | 92.19 | 97.48 | 94.84 | 28.26 | 28.53 | 28.40 | 0.30 | 2.41E-24 |
| UN063352 | Cytochrome P450 family 72 protein | 90.35 | 95.01 | 92.68 | 28.28 | 28.40 | 28.34 | 0.31 | 1.01E-24 |
| UN063353 | Cytochrome P450 family 72 protein | 51.74 | 60.73 | 56.23 | 13.08 | 14.97 | 14.03 | 0.25 | 1.37E-10 |
| UN063354 | Cytochrome P450 family 72 protein | 67.72 | 73.52 | 70.62 | 19.28 | 20.19 | 19.73 | 0.28 | 4.10E-19 |
| UN069645 | Cytochrome P450 family 72 protein | 65.16 | 65.07 | 65.11 | 20.34 | 26.25 | 23.30 | 0.36 | 5.55E-20 |
| UN080568 | Cytochrome P450 family 72 protein | 0.00 | 0.00 | 0.00 | 0.39 | 0.25 | 0.32 | 32.00 | 6.51E-01 |
| UN001980 | Cytochrome P450 family protein | 0.00 | 0.00 | 0.00 | 2.46 | 2.53 | 2.50 | 249.50 | 3.95E-10 |
| UN002459 | Cytochrome P450 family protein | 11.70 | 10.18 | 10.94 | 3.73 | 6.11 | 4.92 | 0.45 | 1.11E-09 |
| UN003946 | Cytochrome P450 family protein | 2.08 | 1.22 | 1.65 | 1.54 | 1.61 | 1.57 | 0.95 | 5.15E-01 |
| UN011612 | Cytochrome P450 family protein | 5.03 | 4.09 | 4.56 | 2.88 | 3.19 | 3.04 | 0.67 | 2.77E-03 |
| UN014840 | Cytochrome P450 family protein | 44.12 | 42.42 | 43.27 | 25.83 | 31.88 | 28.85 | 0.67 | 2.03E-05 |
| UN016557 | Cytochrome P450 family protein | 1.61 | 1.30 | 1.46 | 0.22 | 0.43 | 0.33 | 0.22 | 4.58E-02 |
| UN020781 | Cytochrome P450 family protein | 8.97 | 16.09 | 12.53 | 1.25 | 2.94 | 2.09 | 0.17 | 1.30E-02 |
| UN024707 | Cytochrome P450 family protein | 1.27 | 0.76 | 1.02 | 0.71 | 0.58 | 0.65 | 0.64 | 3.29E-01 |
| UN024708 | Cytochrome P450 family protein | 0.33 | 0.00 | 0.17 | 0.25 | 0.00 | 0.12 | 0.76 | 1.00E+00 |
| UN026257 | Cytochrome P450 family protein | 21.42 | 20.05 | 20.73 | 4.18 | 4.35 | 4.26 | 0.21 | 2.65E-30 |
| UN027314 | Cytochrome P450 family protein | 0.19 | 0.05 | 0.12 | 5.23 | 6.32 | 5.78 | 48.13 | 1.67E-34 |
| UN029300 | Cytochrome P450 family protein | 0.37 | 0.00 | 0.18 | 0.00 | 0.00 | 0.00 | 0.05 | 7.74E-01 |
| UN029302 | Cytochrome P450 family protein | 0.74 | 0.16 | 0.45 | 0.00 | 0.13 | 0.07 | 0.14 | 4.51E-01 |
| UN032436 | Cytochrome P450 family protein | 7.67 | 8.30 | 7.99 | 10.85 | 10.94 | 10.89 | 1.36 | 7.49E-01 |
| UN032438 | Cytochrome P450 family protein | 7.22 | 7.58 | 7.40 | 10.20 | 10.12 | 10.16 | 1.37 | 7.40E-01 |
| UN032440 | Cytochrome P450 family protein | 7.32 | 7.50 | 7.41 | 10.34 | 10.04 | 10.19 | 1.38 | 7.37E-01 |
| UN037122 | Cytochrome P450 family protein | 0.31 | 0.60 | 0.45 | 2.26 | 1.17 | 1.71 | 3.77 | 3.87E-01 |
| UN039618 | Cytochrome P450 family protein | 0.61 | 0.53 | 0.57 | 3.95 | 2.92 | 3.44 | 6.03 | 6.08E-04 |
| UN051357 | Cytochrome P450 family protein | 1.74 | 2.13 | 1.94 | 1.94 | 1.35 | 1.65 | 0.85 | 7.48E-01 |
| UN071183 | Cytochrome P450 family protein | 17.02 | 11.89 | 14.46 | 9.98 | 10.67 | 10.32 | 0.71 | 3.30E-03 |
| UN083421 | Cytochrome P450 family protein | 0.00 | 0.18 | 0.09 | 0.97 | 0.49 | 0.73 | 8.11 | 5.50E-02 |
| UN088570 | Cytochrome P450 family protein | 0.43 | 0.55 | 0.49 | 0.32 | 0.31 | 0.32 | 0.64 | 9.29E-01 |
| UN090279 | Cytochrome P450 family protein | 0.00 | 0.36 | 0.18 | 0.00 | 0.75 | 0.38 | 2.08 | 1.00E+00 |
| UN092859 | Cytochrome P450 family protein | 2.47 | 2.23 | 2.35 | 34.79 | 25.91 | 30.35 | 12.91 | 2.07E-11 |
| UN094258 | Cytochrome P450 family protein | 0.28 | 0.37 | 0.33 | 0.21 | 0.40 | 0.30 | 0.94 | 1.00E+00 |
| UN100582 | Cytochrome P450 family protein | 1.92 | 1.76 | 1.84 | 0.41 | 0.59 | 0.50 | 0.27 | 6.83E-06 |
| UN101045 | Cytochrome P450 family protein | 10.74 | 9.06 | 9.90 | 2.66 | 3.34 | 3.00 | 0.30 | 2.39E-15 |
| UN101055 | Cytochrome P450 family protein | 0.19 | 0.25 | 0.22 | 0.32 | 0.81 | 0.57 | 2.57 | 8.14E-01 |
| UN040333 | Cytochrome P450 protein | 0.08 | 0.02 | 0.05 | 3.60 | 4.77 | 4.18 | 83.70 | 1.43E-24 |
| UN078302 | Cytochrome P450 protein | 0.81 | 0.52 | 0.67 | 0.00 | 0.00 | 0.00 | 0.02 | 4.52E-02 |
| UN082005 | Cytochrome P450 protein | 0.00 | 0.00 | 0.00 | 0.95 | 1.53 | 1.24 | 124.00 | 5.71E-02 |
| UN002137 | Cytochrome P450 superfamily protein | 1.76 | 1.93 | 1.85 | 1.78 | 1.77 | 1.77 | 0.96 | 7.95E-01 |
| UN020368 | Cytochrome P450 superfamily protein | 0.94 | 0.81 | 0.88 | 0.53 | 0.84 | 0.69 | 0.78 | 9.24E-01 |
| UN083456 | Cytochrome P450 superfamily protein | 2.75 | 2.30 | 2.52 | 0.99 | 1.01 | 1.00 | 0.40 | 2.93E-05 |
| UN000266 | Cytochrome P450, putative | 0.17 | 0.43 | 0.30 | 0.00 | 0.00 | 0.00 | 0.03 | 7.26E-01 |
| UN008801 | Cytochrome P450, putative | 1.29 | 1.26 | 1.27 | 6.03 | 7.43 | 6.73 | 5.28 | 3.91E-13 |
| UN011062 | Cytochrome P450, putative | 1.72 | 1.69 | 1.71 | 7.56 | 7.77 | 7.67 | 4.50 | 3.88E-15 |
| UN011978 | Cytochrome P450, putative | 0.47 | 0.76 | 0.61 | 0.13 | 0.25 | 0.19 | 0.31 | 5.35E-01 |
| UN012195 | Cytochrome P450, putative | 0.59 | 0.88 | 0.73 | 0.54 | 0.21 | 0.38 | 0.51 | 6.17E-01 |
| UN013038 | Cytochrome P450, putative | 0.05 | 0.03 | 0.04 | 0.70 | 0.90 | 0.80 | 20.00 | 2.04E-04 |
| UN013039 | Cytochrome P450, putative | 0.06 | 0.00 | 0.03 | 0.26 | 0.38 | 0.32 | 10.67 | 4.45E-01 |
| UN015260 | Cytochrome P450, putative | 5.29 | 6.62 | 5.96 | 3.03 | 2.82 | 2.92 | 0.49 | 1.21E-03 |
| UN016254 | Cytochrome P450, putative | 0.06 | 0.00 | 0.03 | 0.19 | 0.80 | 0.49 | 16.50 | 3.39E-01 |
| UN016559 | Cytochrome P450, putative | 1.61 | 2.91 | 2.26 | 0.41 | 0.27 | 0.34 | 0.15 | 1.10E-02 |
| UN019768 | Cytochrome P450, putative | 0.66 | 1.14 | 0.90 | 0.12 | 0.00 | 0.06 | 0.07 | 5.92E-02 |
| UN020398 | Cytochrome P450, putative | 256.58 | 255.07 | 255.82 | 50.04 | 73.02 | 61.53 | 0.24 | 1.19E-34 |
| UN023462 | Cytochrome P450, putative | 1.66 | 1.15 | 1.41 | 0.32 | 0.17 | 0.24 | 0.17 | 9.52E-05 |
| UN024058 | Cytochrome P450, putative | 2.26 | 2.05 | 2.15 | 0.29 | 0.14 | 0.21 | 0.10 | 9.09E-13 |
| UN024085 | Cytochrome P450, putative | 0.00 | 0.00 | 0.00 | 0.00 | 0.20 | 0.10 | 10.00 | 1.00E+00 |
| UN026258 | Cytochrome P450, putative | 24.04 | 22.75 | 23.39 | 4.73 | 4.97 | 4.85 | 0.21 | 9.86E-31 |
| UN026882 | Cytochrome P450, putative | 0.05 | 0.00 | 0.03 | 2.34 | 1.86 | 2.10 | 84.00 | 8.15E-14 |
| UN030082 | Cytochrome P450, putative | 22.18 | 17.87 | 20.02 | 3.27 | 4.76 | 4.01 | 0.20 | 3.66E-26 |
| UN030083 | Cytochrome P450, putative | 14.74 | 11.82 | 13.28 | 2.53 | 3.30 | 2.92 | 0.22 | 1.61E-23 |
| UN033666 | Cytochrome P450, putative | 14.30 | 13.55 | 13.93 | 10.37 | 10.35 | 10.36 | 0.74 | 1.78E-03 |
| UN033667 | Cytochrome P450, putative | 15.20 | 14.15 | 14.68 | 11.39 | 11.31 | 11.35 | 0.77 | 4.36E-03 |
| UN033668 | Cytochrome P450, putative | 47.06 | 41.77 | 44.42 | 17.24 | 18.94 | 18.09 | 0.41 | 8.83E-16 |
| UN033669 | Cytochrome P450, putative | 0.35 | 0.15 | 0.25 | 2.35 | 2.39 | 2.37 | 9.48 | 1.93E-02 |
| UN036317 | Cytochrome P450, putative | 23.06 | 34.31 | 28.69 | 26.44 | 23.47 | 24.95 | 0.87 | 5.73E-01 |
| UN036318 | Cytochrome P450, putative | 19.86 | 33.63 | 26.75 | 39.12 | 33.75 | 36.44 | 1.36 | 9.13E-01 |
| UN042280 | Cytochrome P450, putative | 0.71 | 1.84 | 1.27 | 0.59 | 0.38 | 0.48 | 0.38 | 5.01E-01 |
| UN042481 | Cytochrome P450, putative | 0.18 | 0.08 | 0.13 | 0.72 | 1.07 | 0.90 | 6.88 | 9.17E-02 |
| UN046249 | Cytochrome P450, putative | 0.26 | 0.00 | 0.13 | 0.75 | 0.84 | 0.79 | 6.12 | 1.42E-01 |
| UN046251 | Cytochrome P450, putative | 0.32 | 0.27 | 0.30 | 1.39 | 1.20 | 1.29 | 4.39 | 5.83E-03 |
| UN049499 | Cytochrome P450, putative | 16.67 | 20.85 | 18.76 | 4.23 | 5.16 | 4.70 | 0.25 | 4.91E-07 |
| UN049500 | Cytochrome P450, putative | 20.02 | 25.32 | 22.67 | 4.90 | 5.12 | 5.01 | 0.22 | 2.49E-07 |
| UN052089 | Cytochrome P450, putative | 0.27 | 0.00 | 0.14 | 0.46 | 0.58 | 0.52 | 3.85 | 8.64E-01 |
| UN053840 | Cytochrome P450, putative | 2.17 | 2.72 | 2.44 | 0.80 | 1.01 | 0.91 | 0.37 | 8.04E-05 |
| UN053841 | Cytochrome P450, putative | 0.20 | 0.19 | 0.20 | 0.88 | 1.12 | 1.00 | 5.13 | 7.68E-02 |
| UN053842 | Cytochrome P450, putative | 0.70 | 1.07 | 0.89 | 0.75 | 1.11 | 0.93 | 1.05 | 9.66E-01 |
| UN053843 | Cytochrome P450, putative | 2.70 | 3.37 | 3.04 | 0.83 | 1.01 | 0.92 | 0.30 | 6.45E-06 |
| UN053844 | Cytochrome P450, putative | 1.48 | 1.53 | 1.50 | 0.49 | 0.53 | 0.51 | 0.34 | 1.31E-02 |
| UN053845 | Cytochrome P450, putative | 1.22 | 1.72 | 1.47 | 0.78 | 1.11 | 0.95 | 0.64 | 1.67E-01 |
| UN055641 | Cytochrome P450, putative | 12.86 | 11.84 | 12.35 | 6.18 | 6.86 | 6.52 | 0.53 | 6.30E-08 |
| UN055642 | Cytochrome P450, putative | 13.86 | 13.32 | 13.59 | 6.67 | 7.54 | 7.11 | 0.52 | 2.98E-08 |
| UN059924 | Cytochrome P450, putative | 1.45 | 1.32 | 1.39 | 6.11 | 7.65 | 6.88 | 4.97 | 7.20E-12 |
| UN059925 | Cytochrome P450, putative | 2.51 | 2.07 | 2.29 | 10.71 | 12.95 | 11.83 | 5.17 | 5.12E-16 |
| UN059926 | Cytochrome P450, putative | 2.23 | 1.98 | 2.10 | 9.86 | 11.68 | 10.77 | 5.12 | 9.17E-15 |
| UN059927 | Cytochrome P450, putative | 1.72 | 1.56 | 1.64 | 7.85 | 9.39 | 8.62 | 5.26 | 3.95E-16 |
| UN060690 | Cytochrome P450, putative | 0.38 | 0.39 | 0.39 | 6.05 | 7.87 | 6.96 | 18.08 | 8.22E-21 |
| UN060701 | Cytochrome P450, putative | 0.21 | 0.00 | 0.10 | 3.49 | 3.69 | 3.59 | 34.19 | 1.44E-05 |
| UN070983 | Cytochrome P450, putative | 0.35 | 0.79 | 0.57 | 6.88 | 6.73 | 6.80 | 11.94 | 5.72E-23 |
| UN070984 | Cytochrome P450, putative | 0.30 | 0.85 | 0.57 | 10.22 | 10.14 | 10.18 | 17.70 | 2.52E-35 |
| UN070987 | Cytochrome P450, putative | 1.22 | 1.69 | 1.46 | 4.13 | 4.81 | 4.47 | 3.07 | 1.36E-03 |
| UN070988 | Cytochrome P450, putative | 2.44 | 3.76 | 3.10 | 3.04 | 4.56 | 3.80 | 1.23 | 1.00E+00 |
| UN070989 | Cytochrome P450, putative | 0.50 | 0.81 | 0.66 | 5.28 | 5.71 | 5.50 | 8.39 | 9.65E-11 |
| UN070993 | Cytochrome P450, putative | 0.33 | 0.00 | 0.17 | 0.18 | 0.52 | 0.35 | 2.12 | 1.00E+00 |
| UN071182 | Cytochrome P450, putative | 10.98 | 8.43 | 9.71 | 9.13 | 10.41 | 9.77 | 1.01 | 5.68E-01 |
| UN071184 | Cytochrome P450, putative | 11.31 | 8.56 | 9.94 | 9.62 | 11.02 | 10.32 | 1.04 | 6.05E-01 |
| UN073268 | Cytochrome P450, putative | 0.56 | 0.36 | 0.46 | 0.93 | 0.40 | 0.67 | 1.45 | 1.00E+00 |
| UN073269 | Cytochrome P450, putative | 0.00 | 0.00 | 0.00 | 0.00 | 0.00 | 0.00 | 1.00 | NA |
| UN073272 | Cytochrome P450, putative | 0.32 | 0.14 | 0.23 | 0.36 | 0.58 | 0.47 | 2.04 | 1.00E+00 |
| UN075939 | Cytochrome P450, putative | 0.00 | 0.00 | 0.00 | 5.58 | 7.14 | 6.36 | 636.00 | 2.48E-39 |
| UN076041 | Cytochrome P450, putative | 3.75 | 2.94 | 3.34 | 3.48 | 3.17 | 3.33 | 0.99 | 5.02E-01 |
| UN076238 | Cytochrome P450, putative | 738.50 | 724.56 | 731.53 | 124.93 | 239.45 | 182.19 | 0.25 | 8.14E-33 |
| UN079385 | Cytochrome P450, putative | 0.00 | 0.00 | 0.00 | 0.00 | 0.18 | 0.09 | 9.00 | 1.00E+00 |
| UN080469 | Cytochrome P450, putative | 0.00 | 0.00 | 0.00 | 0.00 | 0.00 | 0.00 | 1.00 | NA |
| UN080632 | Cytochrome P450, putative | 60.71 | 48.94 | 54.83 | 62.61 | 78.14 | 70.38 | 1.28 | 1.00E+00 |
| UN083251 | Cytochrome P450, putative | 1.60 | 0.93 | 1.27 | 13.83 | 11.19 | 12.51 | 9.89 | 1.56E-14 |
| UN083291 | Cytochrome P450, putative | 2.28 | 3.70 | 2.99 | 1.74 | 2.73 | 2.23 | 0.75 | 5.38E-01 |
| UN084126 | Cytochrome P450, putative | 5.74 | 4.62 | 5.18 | 9.35 | 4.96 | 7.15 | 1.38 | 9.96E-01 |
| UN084447 | Cytochrome P450, putative | 0.00 | 0.41 | 0.20 | 0.00 | 0.00 | 0.00 | 0.05 | 8.97E-01 |
| UN085779 | Cytochrome P450, putative | 0.24 | 0.52 | 0.38 | 0.90 | 0.44 | 0.67 | 1.76 | 9.29E-01 |
| UN087935 | Cytochrome P450, putative | 0.14 | 0.36 | 0.25 | 0.00 | 0.00 | 0.00 | 0.04 | 7.26E-01 |
| UN089079 | Cytochrome P450, putative | 0.03 | 0.07 | 0.05 | 3.38 | 3.64 | 3.51 | 70.20 | 1.21E-21 |
| UN089154 | Cytochrome P450, putative | 38.89 | 40.83 | 39.86 | 14.71 | 15.01 | 14.86 | 0.37 | 1.42E-17 |
| UN089640 | Cytochrome P450, putative | 69.75 | 61.54 | 65.64 | 45.01 | 46.74 | 45.88 | 0.70 | 4.78E-05 |
| UN091173 | Cytochrome P450, putative | 309.82 | 317.66 | 313.74 | 73.94 | 102.50 | 88.22 | 0.28 | 6.04E-29 |
| UN097965 | Cytochrome P450, putative | 0.59 | 0.19 | 0.39 | 0.33 | 0.16 | 0.24 | 0.63 | 9.29E-01 |
| UN100778 | Cytochrome P450, putative | 2.21 | 4.30 | 3.25 | 1.39 | 0.84 | 1.11 | 0.34 | 1.50E-01 |
| UN089854 | 1-deoxy-D-xylulose 5-phosphate synthase | 100.37 | 103.06 | 101.72 | 29.85 | 32.06 | 30.96 | 0.30 | 1.09E-25 |
| UN008472 | 1-deoxy-D-xylulose 5-phosphate reductoisomerase | 52.08 | 53.59 | 52.84 | 23.28 | 23.98 | 23.63 | 0.45 | 1.42E-13 |
| UN058091 | 1-deoxy-D-xylulose 5-phosphate reductoisomerase | 15.88 | 15.68 | 15.78 | 38.13 | 37.60 | 37.87 | 2.40 | 3.69E-06 |
| UN058092 | 1-deoxy-D-xylulose 5-phosphate reductoisomerase | 5.26 | 6.70 | 5.98 | 43.07 | 48.58 | 45.83 | 7.66 | 2.44E-25 |
| UN058348 | 2-C-methyl-D-erythritol 4-phosphate cytidylyltransferase | 18.43 | 19.57 | 19.00 | 10.78 | 11.34 | 11.06 | 0.58 | 4.05E-06 |
| UN058349 | 2-C-methyl-D-erythritol 4-phosphate cytidylyltransferase | 21.83 | 24.09 | 22.96 | 12.71 | 13.52 | 13.12 | 0.57 | 1.04E-06 |
| UN084591 | 4-diphosphocytidyl-2-C-methyl-D-erythritol kinase | 0.42 | 1.08 | 0.75 | 0.47 | 0.15 | 0.31 | 0.41 | 6.54E-01 |
| UN008708 | 4-hydroxy-3-methylbut-2-enyl diphosphate reductase | 74.65 | 82.73 | 78.69 | 26.04 | 30.52 | 28.28 | 0.36 | 6.34E-12 |
| UN069101 | 4-hydroxy-3-methylbut-2-en-1-yl diphosphate synthase (flavodoxin) | 57.48 | 48.85 | 53.16 | 21.06 | 24.52 | 22.79 | 0.43 | 3.92E-15 |
| UN069102 | 4-hydroxy-3-methylbut-2-en-1-yl diphosphate synthase (flavodoxin) | 63.86 | 53.89 | 58.88 | 23.12 | 27.03 | 25.08 | 0.43 | 2.13E-15 |
| UN069103 | 4-hydroxy-3-methylbut-2-en-1-yl diphosphate synthase (flavodoxin) | 59.27 | 50.27 | 54.77 | 21.64 | 25.10 | 23.37 | 0.43 | 2.91E-15 |
| UN069104 | 4-hydroxy-3-methylbut-2-en-1-yl diphosphate synthase (flavodoxin) | 46.13 | 37.13 | 41.63 | 16.25 | 17.82 | 17.04 | 0.41 | 2.66E-16 |
| UN019173 | 4-hydroxy-3-methylbut-2-en-1-yl diphosphate synthase protein | 0.91 | 0.26 | 0.58 | 0.56 | 1.29 | 0.93 | 1.58 | 1.00E+00 |
| UN019174 | 4-hydroxy-3-methylbut-2-en-1-yl diphosphate synthase protein | 0.78 | 0.54 | 0.66 | 0.75 | 1.06 | 0.91 | 1.37 | 1.00E+00 |
| UN025397 | 4-hydroxy-3-methylbut-2-enyl diphosphate reductase | 0.17 | 0.22 | 0.20 | 0.19 | 0.00 | 0.10 | 0.49 | 1.00E+00 |
| UN025399 | 4-hydroxy-3-methylbut-2-enyl diphosphate reductase | 0.19 | 0.00 | 0.10 | 0.63 | 0.40 | 0.52 | 5.42 | 6.23E-01 |
| UN025400 | 4-hydroxy-3-methylbut-2-enyl diphosphate reductase | 0.23 | 0.15 | 0.19 | 0.39 | 0.38 | 0.39 | 2.03 | 1.00E+00 |
| UN025401 | 4-hydroxy-3-methylbut-2-enyl diphosphate reductase | 0.54 | 0.10 | 0.32 | 0.77 | 0.75 | 0.76 | 2.38 | 7.96E-01 |
| UN059389 | 4-hydroxy-3-methylbut-2-enyl diphosphate reductase | 15.92 | 20.56 | 18.24 | 8.10 | 10.74 | 9.42 | 0.52 | 8.50E-03 |
| UN059391 | 4-hydroxy-3-methylbut-2-enyl diphosphate reductase | 3.60 | 3.92 | 3.76 | 2.28 | 3.92 | 3.10 | 0.82 | 3.65E-01 |
| UN059392 | 4-hydroxy-3-methylbut-2-enyl diphosphate reductase | 45.92 | 44.82 | 45.37 | 21.01 | 27.62 | 24.32 | 0.54 | 2.94E-09 |
| UN025398 | 4-hydroxy-3-methylbut-2-enyl diphosphate reductase, putative | 0.36 | 0.00 | 0.18 | 0.20 | 0.00 | 0.10 | 0.56 | 1.00E+00 |
| UN059390 | 4-hydroxy-3-methylbut-2-enyl diphosphate reductase, putative | 5.20 | 5.76 | 5.48 | 10.71 | 13.14 | 11.93 | 2.18 | 4.77E-03 |
