# Supplementary material for: NAA and 6-BA promote accumulation of oleanolic acid by JA regulation in Achyranthes bidentata Bl
Source: PLoS One. 2020 Feb 27;15(2):e0229490. doi: 10.1371/journal.pone.0229490 (PMC7046271; doi:10.1371/journal.pone.0229490)
Supplement: S5 Table — The CYP450s marked in red represent those used in the construction of Fig 5. (DOCX) [file pone.0229490.s009.docx]

**Table S5. List of fifty-five CYP450s related to terpenoid biosynthesis.**

| Organism | Gene name | NCBI Accession |
| --- | --- | --- |
| *Platycodon grandiflorus* | CYP716S6 | AOG74840.1 |
|  | CYP716S4 | AOG74837.1 |
|  | CYP72A556 | BAX04012.1 |
|  | CYP72A555 | BAX04011.1 |
|  | CYP72A554 | BAX04010.1 |
|  | CYP716D58 | BAX04009.1 |
|  | CYP716A141 | BAX04008.1 |
|  | CYP716A140v2 | BAX04007.1 |
| *Medicago truncatula* | CYP72A65v2 | BAL45202 |
|  | CYP72A63 | H1A981.1 |
|  | CYP72A67v2 | BAL45203 |
|  | CYP72A61v2 | BAL45199 |
|  | CYP716A12 | ABC59076.1 |
|  | CYP72A66v2 | QFS19032.1 |
|  | CYP72A560 | QFS19029.1 |
|  | CYP72A64v2 | QFS19030.1 |
|  | CYP72A559 | QFS19028.1 |
|  | CYP72A558 | QFS19027.1 |
|  | CYP72A557 | QFS19026.1 |
|  | CYP72A337v2 | QFS19026.1 |
|  | CYP72A336v2 | QFS19022.1 |
|  | CYP72A70 | QFS19023.1 |
| *Aquilegia coerulea* | CYP716A111 | APG38190.1 |
| *Glycine max* | CYP72A141 | QFS19014.1 |
| *Maesa lanceolata* | CYP87D16 | AHF22090.1 |
|  | CYP96A69 | AHF22091.1 |
|  | CYP71AU39 | AHF22087.1 |
|  | CYP716A75 | AHF22088.1 |
|  | CYP96A68 | AHF22086.1 |
|  | CYP94A43 | AHF22085.1 |
|  | CYP79D33 | AHF22089.1 |
| *Catharanthus roseus* | CYP71D1V2 | AEX07771.1 |
|  | CYP71D1V1 | AEX07770.1 |
|  | CYP716AL1 | AEX07773.1 |
| *Lotus japonicus* | CYP716A51 | BAP59952.1 |
|  | CYP72A697 | QFS19021.1 |
|  | CYP93E1 | BBF88366.1 |
| *Panax ginseng* | CYP716A47 | H2DH16.2 |
|  | CYP716A53v2 | I7CT85.1 |
|  | CYP716A52v2 | AFO63032.1 |
| *Cajanus cajan* | CYP72A696 | QFS19013.1 |
|  | CYP72A695 | QFS19013.1 |
| *Glycine soja* | CYP72A141 | QFS19024.1 |
| *Phaseolus vulgaris* | CYP72A302 | QFS19019.1 |
| *Vigna angularis* | CYP72A694 | QFS19020.1 |
| *Trifolium pratense* | CYP72A699 | QFS19031.1 |
| *Glycyrrhiza pallidiflora* | CYP72A154 | QFS19016.1 |
| *Glycyrrhiza uralensis* | GuCYP72A154 | H1A988.1 |
|  | GuCYP88D6 | B5BSX1.1 |
| *Avena strigosa* | CYP51H10 | ABG88965.1 |
| *Vitis vinifera* | CYP716A17 | BAJ84107.1 |
|  | CYP716A15 | BAJ84106.1 |
| *Populus trichocarpa* | CYP71B63v2 | AIU56749.1 |
| *Sorghum bicolor* | CYP71AM1 | AVI24670.1 |
|  | CYP71AF1 | AVI24671.1 |
