# Supplementary material for: NAA and 6-BA promote accumulation of oleanolic acid by JA regulation in Achyranthes bidentata Bl
Source: PLoS One. 2020 Feb 27;15(2):e0229490. doi: 10.1371/journal.pone.0229490 (PMC7046271; doi:10.1371/journal.pone.0229490)
Supplement: S6 Table — The unigenes marked in red represent those used in the construction of Fig 5. (DOCX) [file pone.0229490.s010.docx]

**Table S6. List of twenty-two unigenes of *A. bidentata* used to build Phylogenetic tree.**

| Unigene number | GenBank accession | Unigene number | GenBankaccession |
| --- | --- | --- | --- |
| UN008801 | MN732950 | UN075939 | MN732961 |
| UN026882 | MN732951 | UN089079 | MN732962 |
| UN036441 | MN732952 | UN093792 | MN732963 |
| UN040333 | MN732953 | UN082587 | MN732964 |
| UN046251 | MN732954 | UN085763 | MN732965 |
| UN059924 | MN732955 | UN003649 | MN732966 |
| UN059925 | MN732956 | UN013038 | MN732967 |
| UN059926 | MN732957 | UN046523 | MN732968 |
| UN059927 | MN732958 | UN016253 | MN732969 |
| UN070983 | MN732959 | UN027314 | MN732970 |
| UN070984 | MN732960 | UN092859 | MN732971 |
