# Supplementary material for: NAA and 6-BA promote accumulation of oleanolic acid by JA regulation in Achyranthes bidentata Bl
Source: PLoS One. 2020 Feb 27;15(2):e0229490. doi: 10.1371/journal.pone.0229490 (PMC7046271; doi:10.1371/journal.pone.0229490)
Supplement: Attachment — Submitted filename: Response to Reviewers.docx [file pone.0229490.s011.docx]

## Response to Reviewers

Dear editor and reviewers:

We would like to thank PLOS ONE for giving us opportunity to revise our manuscript. We thank the reviewers for their carefully reading and thoughtful comments on previous draft. We have carefully taken their comments into consideration in preparing our revision, which has resulted in the paper that is clearer, more compelling. Below is our response to their comments. Thanks for all the help. Best wishes.

### Responds to academic editor:

1. I have carefully checked the manuscript, and the manuscript has met PLOS ONE's style requirements, including those for file naming.
2. I have modified the manuscript for language usage, spelling, and grammar and sincerely hope that it can meet the requirements of the magazine.

My colleague, Li Tang, and I participated in the revision of the manuscript,and my name is Yanqing Liu.

For the resubmission, I have provide the following files:

“Response to Reviewers”;

“Revised Manuscript with Track Changes” (uploaded as a *supporting information* file) and a clean copy of the edited manuscript (uploaded as the new *manuscript* file).

3. In my Methods section, I have provided additional details regarding the *A. bidentata* seeds used in the study.
4. In the revised cover letter, I have provided the relevant accession numbers that may be used to access data. All the readings obtained in this study have been uploaded to the NCBI Sequence Read Archive, and the accession number is PRJNA350183.

### Responds to reviewers:

Reviewer #1

1/ Lines 47-50

I have added the following references:

Miettinen K, Iñigo S, Kreft L, Pollier J, De Bo C, Botzki A, et al. The TriForC database: a comprehensive up-to-date resource of plant triterpene biosynthesis. Nucleic Acids Res. 2018; 46(D1): D586-D594. [https://doi.org/10.1093/nar/gkx925](https://doi.org/10.1093/nar/gkx925.) PMID:PMC5753214

2/ Lines 52-54

The previous paper was incorrectly stated. I have changed the content of the paper that “The cyclization of 2,3-oxidosqualene catalyzed by OSCs is a key step in the biosynthesis of triterpenoid saponins and sterols”.

3/ Line 56

I have mistakenly spelled before, and have already changed the word “Cholesterol” to “phytosterol”.

4/ Lines 87-89 (changed to lines 87-98)

A more detailed statement about the JA signaling mechanism is :

In the presence of JA-isoleucine, the SCF^COI1^ complex forms, and JAZ proteins are degraded by the 26S proteasome. The ubiquitination of JAZ protein binds to substrate-specific SCF^COI1^, and the degradation of JAZ protein by the 26S proteasome abolishes this interaction. The JAZ proteins contain a conserved TIFY motif within the ZIM domain that mediates homoand hetero-dimeric interactions between different JAZ proteins. The ZIM domain also functions to recruit transcriptional corepressors through the novel interactor of JAZ protein. The JAZ proteins contain Jas domain that is required for the interaction of both COI1 and a broad array of TFs. In the presence of JA or its bioactive derivatives, JAZ proteins are degraded and freeing TFs for expression of specific sets of JA-responsive genes, regulate enzymes involved in the biosynthesis of secondary metabolites, such as ginsenoside, artemisinin, and vinblastine.

5/ Lines 104-107 (changed to lines 112-115)

The detailed treatment of plants is described below.

After 20 days of emergence, the leaves of plants were spraied with a mixture of different concentrations of NAA and 6-BA until there was liquid dripping at the edge of the blade. The mixture included: 1.0 mg/L NAA + 0.5 mg/L 6-BA (T1); 2.0 mg/L NAA + 1.0 mg/L 6-BA (T2); 4.0 mg/L NAA + 2.0 mg/L 6-BA (T3) and 8.0 mg/L NAA + 4.0 mg/L 6-BA (T4).

6/ Lines 126-128 (changed to lines 136-139)

The method for determining the chlorophyll (Chl. a, Chl. b) and carotenoid contents is expressed as follows:

The second pair of leaves from the top of plants (Control and T2 group) was harvested to measure the contents of chlorophyll a, b, and carotenoid. Fresh chopped leaves (0.1 g) added in 10 mL of 80% acetone (v/v) at room temperature in a dark environment for 24 h, and then measured the absorbance at 645 nm, 663 nm and 470 nm, respectively, for detecting chlorophyll a, b and carotenoids contents.

7/ Lines 252-254 (changed to lines 262-264)

Some genes indeed are part of different processes. Many of the genes in *A. bidentata* treated with NAA and 6-BA were affected because the various biological processes are complex and some genes are involved in other biological reaction process. Therefore, the percentage of different processes in the GO analysis is very high.

8/ Lines 297-299 (changed to lines 312-314)

About 28% of the 900 species in the Amaranthaceae estimated to occur in the family are C4 species, such as *Gomphrena meyeniana*, *Amaranthus hypochondriacus.*

The references is:

Sage RF, Sage TL, Pearcy RW, Borsch T. The taxonomic distribution of C4 photosynthesis in *Amaranthaceae sensu* stricto. Am J Bot. 2007; 94(12): 1992-2003. <https://doi.org/10.3732/ajb.94.12.1992> PMID: 21636394

9/ Lines 313-314 (changed to lines 328-329)

After the modification, I have used bZIP and bHLH as abbreviation.

10/ Lines 346-347 (changed to lines 361-362)

The results are the data analysis we have done. The production of glycyrrhizin is oxidized at the C-11 position while oleanolic acid biosynthesis is at C-28 position oxidized. It may involved in ent-kaurene biosynthesis, because we observed that the ent-laurene synthase gene is up-regulated in the biosynthesis of gibberellin.

11/ Lines 347-349 (changed to lines 362-364)

The saponins currently isolated and identified from *A. bidentata* are all oleanane triterpenoid saponins. UGT can add sugar chains to oleanolic acid to form oleanane-type triterpenoid saponins, and UGT gene expression is up-regulated, which proves that more oleanane-type triterpenoid saponins are produced. From the side, it is indicated that the content of oleanolic acid is increased. So we analyzed the UGT slightly here.

12/ Remark Figure 4.

Your comments are meaningful, but the data we analyze does have some differences from your point of view. We also selected some genes for qRT-PCR validation, and the results were consistent with the expression pattern of RNA-seq assay. This result may be due to some differences in the expression of some enzyme genes in different species in MVA and MEP pathways.

Ginsenoside biosynthesis is part of our data analysis. In addition, ginsenosides have been reported in the achyranthes. Ginsenosides belong to the triterpenoids, one is the oleanane type pentacyclic triterpenoid saponin Ro, and the other two are the ginsengdiol type saponins (such as Rb1, Rb2, Rc, Rd, F2, Rg3, Rh2, etc.) and Ginseng triol saponins (such as Re, Rg1, Rg2, Rf, Rh1, etc.), both of which belong to the dammarane type tetracyclic triterpenoid saponins.

13/ Remark Figure 5

We used DEGs to analyze the gene data between samples, and analyzed the key enzymes involved in the synthesis of triterpenoid saponins. It was found that there were more differential genes involved in the synthesis of triterpenoid carbon skeleton, and 38 were co-expressed in control roots and treated leaves up-regulated CYP450s gene sequence. In order to further narrow the potential range of CYP450s involved in biosynthesis of oleanolic acid saponin, 25 up-regulated CYP450s (with sequence lengths > 1500 bp) were selected for phylogenetic analysis from *A. bidentata* treated leaves.

14/ Line 374 (changed to lines 389)

Your comment makes a lot of sense, we also consulted a lot of literature: more experiments have shown that MYC, MYB, WRKY and AP2/ERF transcription factors are involved in the regulation of terpene metabolism in different medicinal plants [1, 2]. For example, AaWRKY1, TSAR1, and TSAR2 are found to regulate the expression of HMGR genes in the MVA pathway in *ArtemisiaannuaL.* and *Medicago truncatula.* TSAR1 and TSAR2 are two transcription factors that are induced by jasmonic acid in the bHLH family [3, 4]. In the *Medicago truncatula* hairy roots, overexpression of TSAR1 and TSAR2 significantly increased the transcriptional expression levels of the MVA pathway gene and a series of genes involved in the synthesis of β-amyrin, such as HMGS, HMGRI, SS and β-AS [4, 5]. In our study, most AP2/ERF TFs, BHLHTFS and MYB TFs were up-regulated in treated leaves, and significantly increased the expression of triterpenoid biosynthesis genes in *A. bidentata*, especially HMGR, SS, SE and β-AS gene. Therefore, JA regulates oleanolic acid accumulation by freeing TFs and increasing expression of terpenoid biosynthesis genes in *A. bidentata*. These results were consistent with overexpression of TSARs to promote oleanane-type triterpene saponin in *M. truncatula*.

References:

[1] Afrin S, Jing-Jia H, [Zhi-Yong L](http://xueshu.baidu.com/s?wd=author:(Zhi-Yong Luo) &tn=SE_baiduxueshu_c1gjeupa&ie=utf-8&sc_f_para=sc_hilight=person" \t "http://xueshu.baidu.com/usercenter/paper/_blank). JA-mediated transcriptional regulation of secondary metabolism inmedicinal plants[J]. Science Bulletin. 2015, 60(12): 1062-1072.

[2] Wasternack C, and Hause B. Jasmonates: biosynthesis, perception, signal transduction and action in plant stress response, growth and development. an update to the 2007 review in annals of botany[J]. Annals of Botany. 2013, 111(6): 1021.

[3] Patra B, Schluttenhofer C, Wu Y, et al. Transcriptional regulation of secondary metabolite biosynthesis in plants[J]. Biochimica Et Biophysica Acta. 2013, 1829(11): 1236-1247.

[4] Goossens A, Mertens J, Pollier J, et al. The bHLH Transcription Factors TSAR1 and TSAR2 Regulate Triterpene Saponin Biosynthesis in *Medicago truncatula*[J]. Plant Physiology. 2015, 170(1): 194.

[5] Mertens J, Moerkercke A V, Bossche R V, et al. Clade IVa Basic Helix -Loop -Helix Transcription Factors Form Part of a Conserved Jasmonate Signaling Circuit for the Regulation of Bioactive Plant Terpenoid Boynesis[J]. Plant and Cell Physiology. 2016, 57(12): 2564.

15/ Lines 389-391 (changed to lines 404-406)

We have already done it. (Li Jinting. Effects of Methyl Jasmonate on the Growth and Distribution of Main Medicinal Components in *Achyranthes Bidentata* Blume.)

Spraying 1.0mg/L MeJA could increase the content of oleanolic acid in *A. bidentata*.

16/ Lines 410-411 (changed to lines 425-426)

Spelling mistakes, change tertripenoid to triterpenoid.

Reviewer #2

Line 40

The language expression is not accurate and should be “Oleanolic acid, a pentacyclic triterpenoid, is an isoprenoid-derived compound ”.

Oleanolic acid has been purified during the extraction process.

Preparation of oleanolic acid determination sample: 0.5 g sample was added with methanol 10 mL, ultrasonically extracted for 40 min, and then concentration using a rotary vacuum evaporator. To the concentrated samples 10 mL of 4 mol/L hydrochloric acid was added for hydrolyzing at 85°C for 1 h. After being allowed to cool, 10 mL of chloroform were added to the sample, followed by two countercurrent extractions at 60°C, each extraction being 15 min. The bottom liquid was harvested and evaporated under reduced pressure. Chromatography of pure methanol to a volume of 3 mL, mixed and filtered through a Millipore filter (0.22 μm), and determined by HPLC. The assay conditions were mobile phase: methanol‐water‐glacial acetic acid (90: 10: 0.05); flow rate: 0.9 mL/min; column temperature: 30°C; detection wavelength: 210 nm.

Line 103 (changed to lines 111)

Achyranthes bidentata is one of the famous Dao-di herbs in China, this species is primarily distributed in the Guhuaiqingfu area, located in Jiaozuo in Henan Province. So *A. bidentata* is also called ‘Huainiuxi’. Dao-di herbs mean that natural Chinese medicines that grow naturally under the influence of natural environment and social environment such as specific geography and climate, or are processed and cultivated with good quality and high curative effect and is synonymous with quality medicinal herbs in China. The quality of *A. bidentata* in Jiaozuo is better than in other regions, therefore, we choose to experiment in the natural environment.

Line 202 (changed to lines 212)

Here, it should be compared with the control, these characteristics showed a trend of increasing first and then decreasing. The reason we explore was lower concentrations of NAA and 6-BA promote the growth of *A. bidentata* while higher concentrations inhibited growth of *A. bidentata*. Therefor, it presenting this trend.

Line 204 (changed to lines 214-215)

We have replaced it with a more appropriate expression, using ‘growth parameters’ instead of ‘index’.

Line 257 (changed to lines 267-270)

I have improved the “For the pathway enrichment analysis, a total of 28 pathways were enriched (adjusted P < 0.05) with DEGs (Table 2)” to “To further identify metabolic or signal transduction pathways in which the DEGs are likely to be involved in promoting accumulation of oleanolic acid by JA regulation by NAA and 6-BA treatment in *A. bidentata*, and pathway enrichment analysis was performed using KEGG database. For the pathway enrichment analysis, a total of 28 pathways were enriched (adjusted P < 0.05) with DEGs (Table 2)”.

Line 318 (changed to lines 333)

We identified 22 key enzyme genes involved in the MVA pathway and 23 key enzyme genes involved in the MEP pathway. Comparing the expression in the roots and leaves of *A. bidentata*, it was found that the RPKM values of MCT, HDS and HDR genes were significantly higher than roots. The HMGS, HMGR, PMK and MDD genes in the MVA pathway are highly expressed in roots and low in leaves, which is completely opposite to the expression of key enzyme genes in the MEP pathway. These results are consistent with the expression of key enzyme genes in the MVA pathway and the MEP pathway in different vegetative organs of Astragalus membranaeus Bge. In addition, more documents revealed that protein of CMS, DXS, DXR and HDS enzymes are highly expressed in young roots and floral organs of Arabidopsis. These results clearly demonstrate that the MEP pathway synthesized in Plastid pathway prefers expressed in leaves, while the MVA pathway favors in roots. In root and leaf samples, it is clearly that chloroplasts are common in leaves and are relatively rare in roots. This difference may be due to the fact that the expression of key enzyme genes in the majority of MEP synthesis pathways is significantly higher in leaves than in roots and in the up-regulation of genes in roots in most MVA pathways. The expression patterns of key enzyme genes in different tissues in these two pathways indicated that the regulation of the biosynthetic pathways of IPP and DMAPP in different tissues of *A. bidentata* is parallel but separate.
