# Supplementary material for: NAA and 6-BA promote accumulation of oleanolic acid by JA regulation in Achyranthes bidentata Bl
Source: PLoS One. 2020 Feb 27;15(2):e0229490. doi: 10.1371/journal.pone.0229490 (PMC7046271; doi:10.1371/journal.pone.0229490)
Supplement: Attachment — Submitted filename: Response to Reviewers.docx [file pone.0229490.s012.docx]

## Response to Reviewers

Dear editor and reviewers:

We have tried our best to revise and improve the manuscript and made many changes in the manuscript according to the reviwers′good comments. We appreciate for Editors/Reviewers’ warm work earnestly, and hope that the corrections will meet with approval.

Once again, thank you very much for your comments and suggestions.

We look forward to your information about my revised papers and thank you for your good comments.

**Responds to reviewers:**

Reviewer #1

1/

We have modified the MVA / MEP pathway presented in Figure 4 according to your comments. 2/ Lines 37-39

We have modified the manuscript according to your comments.

3/ Line 54(changed to lines 55)

We have modified it.

4/ Lines 56 (changed to lines 57)

We have modified the manuscript according to your comments

5/ Lines 104-107 (changed to lines 73-89)

We have modified the introduction according to your comments.

6/ Lines 113 (changed to lines 118)

We have modified it to be ‘sprayed’.

7/

We have uploaded sequence information to NCBI. Our records will be released to the public database once they are processed by GenBank staff.

8/

We constructed the ML tree and NJ tree according to your suggestion(At the end of text we provide you with diagrams of NJtree and MLtree.), using 55 P450s related to triterpene synthesis that have been published. It is found that the results of ML tree and NJ tree were similar: UN082587 is classified into the CYP71 subfamily, UN046523 and UN085763 and GuCYP88D6 are highly homologous, and UN003649 and UN013038 are classified into the CYP72 subfamily.

So we chose the NJ tree that many researchers usually use.

The NJ tree we constructed is provided in S4 Fig, and the information of the 55 P450s and 5 unigenes used were shown in S5 Table and S6 Table, respectively. We provide a “SI’’ doc with amino acid sequences of 55 P450s and 5 unigenes, which can be used to build a phylogenetic tree. Information on 22 unigenes and 55 P450s related to triterpene synthesis were provided in the supporting information.

9/

Oleanolic acid synthesis requires oxidation at C-28, while glycyrrhizin is at C-11. So we chose the P450s with the following functions:

11-oxo-beta-amyrin 30-oxidase; involved in the biosynthesis of glycyrrhizin.

Beta-amyrin 28-oxidase.

dammarenediol-II.

Protopanaxatriol synthase.

Beta-amyrin 28-oxidase ;ginsenoside biosynthesis; Oleanic acid synthase.

beta-amyrin 11-oxidase; biosynthesis of the triterpene sweetener glycyrrhizin.

Beta-amyrin 28-oxidase; Multifunctional OxidasesTriterpenoid Biosynthesis.

10/

We found that JA can enhance oleanolic acid accumulationin cell suspension cultures of *Calendula officinalis L.*[Ewa Wiktorowska, Marek Długosz. Significant enhancement of oleanolic acid accumulation by biotic elicitors in cell suspension cultures of Calendula officinalis. Enzyme and Microbial Technology. 2010. ]We have updated in the manuscript.

MeJa and JA are both plant hormones. Due to the aqueous solution of jasmonic acid is very unstable, many articles treat plants with MeJa. At present, many literature were written side by side with JA and MeJa, and MeJa enters plants and is hydrolyzed to JA.

Reviewer #2

1/ Line 37

*A. bidentata* contains free oleanolic acid and its saponins，We extracted the total saponin content in *A. bidentata*, oleanane-type triterpenoid saponins release oleanolic acid by acid hydrolysis. The content of oleanane-type triterpenoid saponins was then determined by measuring the oleanolic acid content. And we have modified the manuscript according to your comments.

2/ Line 130 (changed to lines 148-158)

We have modified the manuscript according to your comments.

3/ Line 211 (changed to lines 230)

*A. bidentata* was treated with four different concentrations of a mixture of NAA and 6-BA. After 20 days , We simultaneously sampled each group and measured these growth parameters separately. With the concentration of the mixture increasing, it presented this trendency. Here, “First increasing then decresing” should be understood as a tendency occurring in concentrations, not in time. We explained T1,T2,T3 and T4 below the legend in the manuscript. And we have modified the figure 1and figure 2 according to your comments.

4/

We have modified it in the manuscript.

**Phylogenetic tree**
