# Supplementary material for: NAA and 6-BA promote accumulation of oleanolic acid by JA regulation in Achyranthes bidentata Bl
Source: PLoS One. 2020 Feb 27;15(2):e0229490. doi: 10.1371/journal.pone.0229490 (PMC7046271; doi:10.1371/journal.pone.0229490)
Supplement: Attachment — Submitted filename: Response to Reviewers.docx [file pone.0229490.s013.docx]

## Response to Reviewers

Dear editor and reviewers:

We have tried our best to revise and improve the manuscript and made many changes in the manuscript according to the reviwers′good comments. We appreciate for editor and reviewers’ warm work earnestly, and hope that the corrections will meet with approval.

Once again, thank you very much for your comments and suggestions.

We look forward to your information about my revised papers and thank you for your good comments.

**Responds to reviewers:**

Reviewer #1

1/

We have modified the manuscript according to your comments.

2/(changed to lines 84)

We have used "alone" to correct "singly".

3/(changed to lines 96)

We have used "homo- and" to correct "homoand"	4/ (changed to lines 136-144)

We have re-written in proper English. And changed to the following:

We weighed 0.5 g dry weight of *A. bidentata* root powder into a triangle bottle (repeated 3 times for each sample group), added 10 mL of methanol to it, sonicated for 40 minutes, and then concentrated using a rotary vacuum evaporator to completely dry the sample. Then 10 mL of 4 mol/L hydrochloric acid was added and hydrolyzed at 85 °C for 1 h. After cooling, 10 mL of chloroform was added to the sample, and then two reflux extractions were performed at 60 °C for 15 minutes each. The lower liquid was collected and concentrated to dryness under reduced pressure. The residue was dissolved in 3 mL of methanol, filtered through a filter (0.22 μm), and measured by HPLC.

5/

We have corrected the order throughout the entire paper. Overall the use of "Table S6" or "Fig S4" instead of "S6 Table" or "S4 Fig".

Reviewer #2

1/lines 37 (changed to lines 38)

We have modified the manuscript according to your comments.

2/lines 225-229 (changed to lines 225-228)

We have modified the manuscript according to your comments.

3/lines 239-240

We have deleted the sentence ''Oleanolic acid content rose at first and then fell” according to your comments.
