# Supplementary material for: NAA and 6-BA promote accumulation of oleanolic acid by JA regulation in Achyranthes bidentata Bl
Source: PLoS One. 2020 Feb 27;15(2):e0229490. doi: 10.1371/journal.pone.0229490 (PMC7046271; doi:10.1371/journal.pone.0229490)
Supplement: Attachment — Submitted filename: Response to Reviewers.docx [file pone.0229490.s014.docx]

## Response to Reviewers

Dear editor and reviewers:

We have tried our best to revise and improve the manuscript and made many changes in the manuscript according to the reviwers′good comments. We appreciate for editor and reviewers’ warm work earnestly, and hope that the corrections will meet with approval.

Once again, thank you very much for your comments and suggestions.

We look forward to your information about my revised papers and thank you for your good comments.

**Responds to reviewers:**

Reviewer #1

1/

We have modified the title according to your comments. We have removed“through de novo transcriptomics”.

2/ (line 28, changed to line 27)

We have modified the manuscript according to your comments. We have changed “oleanolic acid” as “oleanane-type triterpenoid saponins”.

3/ ( line 39)

We have corrected as“possess”. 4/ (Line 52)

We have added a space between “diphosphate” and “synthase”.

5/ (line 57)

We have modified the manuscript according to your comments.

6/ (line 60)

We have changed "are assumed" to "were shown to".

7/ ( line 63-66, changed to line 65)

We have modified the manuscript according to your comments, and changed as “oleanane backbone”.

8/ (line 86, changed to line 85)

We have modified the manuscript according to your comments.

9/ (line 92-95, changed to line 90-96)

We have modified the manuscript according to your comments.

10/ (line 98, changed to line 99)

We have added the abbreviation “NINJA” after “novel interactor of JAZ”.

11/ (line 99)

We have added "a" between “contain” and “Jas”.

12/(line 117, changed to line 118-119)

We have modified the manuscript according to your comments.

13/ (line 140)

We have modified the manuscript according to your comments.

14/ (line 226)

We have corrected to "... the plants 20 days after treatment with..."according to your comments.

15/ ( Legend Fig 2)

We have added a space between “6-BA.” and “Each”.

16/ (line 21-23, changed to line 20-23)

We have corrected it in the abstract.

17/ (Table 4)

We have corrected to “bHLH” and “bZIP”.

18/ ( Legend Figure 4)

We have rechecked the “CMK’’ abbreviation, and no errors found; and removed “50”.

19/ ( Line 419)

We have corrected to “bHLH”.

20/ ( Legend Figure 7)

We have deleted "through de novo sequencing", and changed to “Fig 7. NAA and 6-BA promote accumulation of oleanolic acid by JA regulation in *A. bidentata*.”

21/ (Legend Table S6)

We have changed to "*A. bidentata*".

Reviewer #2

Thank you very much for your comments on the manuscript. Your comments have made the manuscript more clear and reasonable. Thank you again!
